# Fresh Properties, Strength, and Durability of Fiber-Reinforced Geopolymer and Conventional Concrete: A Review

**DOI:** 10.3390/polym16010141

**Published:** 2024-01-01

**Authors:** Osama Mohamed, Haya Zuaiter

**Affiliations:** Department of Civil Engineering, Abu Dhabi University, Abu Dhabi PO Box 59911, United Arab Emirates; 1075529@alumni.adu.ac.ae

**Keywords:** fiber reinforcement, synthetic fibers, natural fibers, alkali-activated binders, fresh properties, mechanical properties, durability properties, fiber bridging, crack propagation

## Abstract

Reducing the environmental footprint of the construction industry in general and concrete in particular is essential. The addition of synthetic and natural fibers to concrete mixes at appropriate dosages enhances durability and strength and extends the lifespan of concrete infrastructures. This study reviews the geometric and mechanical properties of selected fibers such as steel, basalt, polypropylene, polyvinyl alcohol, polyethylene, glass, carbon, and natural fibers and their impact on concrete fresh, mechanical, and durability properties when combined in different configurations. The study focuses on the effect of blending fibers with concrete mixes that use alkali-activated binders based on recycled industrial byproducts such as slag and fly ash and thereby contribute to reduction of CO_2_ contribution through complete or partial replacement of Ordinary Portland cement (OPC). As a result, the effect of binder content, binder composition, alkaline activator concentration, and water-to-binder (w/b) ratio on fresh properties, mechanical strength, and durability of concrete with blended fibers is also evaluated in this study. The properties of fiber-reinforced concrete with alkali-activated binder and conventional OPC binders are compared. Fiber-reinforced concrete with alkali-activated binders that are based on industrial byproducts may represent sustainable alternatives to conventional concrete and offers competitive fresh and mechanical properties when fiber properties, fiber content, w/b ratio, binder type, and dosage are carefully considered in concrete mix design.

## 1. Introduction

Concrete with Ordinary Portland cement (OPC) binder is the most widely used material in the construction industry across the globe. Its high compressive strength, versatility, and relatively low cost made it the preferred choice in various ranges of construction applications. With the rise in urban development, the production of OPC reached four billion metric tons and is projected to increase in the next decade [1]. Producing one ton of cement requires approximately two tons of raw materials and emits nearly equal amounts of carbon dioxide emissions into the atmosphere [2]. The extensive utilization of cement in concrete aggravates global warming with adverse and long-term environmental impact. The utilization of industrial byproducts such fly ash, ground granulated blast furnace slag (GGBS), and silica fume reduces reliance on OPC, enhances concrete properties, and decreases the need for landfills. Other agricultural byproducts, such as rice husk ash, and treated materials such as metakaolin also decrease the reliance on OPC and enhance concrete properties. Concretes with alkali-activated binders mainly depend on the characteristics of the aluminosilicate precursor, the type and concentration of the alkaline activator solution, and the curing environment [3]. All types of concrete, including those with OPC and alkali-activated binders, are characterized mainly by their brittle nature and low tensile strength. These characteristics mainly lead to crack formation, poor durability, and high maintenance cost. Studies found that concretes with industrial byproducts have lower resistance to cracking and higher brittleness compared to OPC-based concretes [4].

The use of fibers as reinforcement in cementitious matrices has become increasingly common. Several studies proposed addressing the brittleness and low tensile strength in cementitious concrete composites by adding fibers. Fibers enhance the ability of concrete structures to withstand relatively higher levels of stress and strain while maintaining structural strength, stability, and durability [5].

The physical interactions between the reinforcing fibers and the concrete matrix enhance the ductility and crack resistance of fiber-reinforced concretes. The strength of the bond between reinforcing fibers and the cementitious matrix is essential to preventing crack propagation [6]. Fibers in concrete composites act as bridges within the concrete matrix through physical bond interactions. The bridging action through reinforcing fibers increases the energy required for a crack to propagate through concrete and enhances its crack resistance [7]. The impact of crack formation and growth on the mechanical properties and durability of concrete is effectively mitigated by the addition of reinforcing fibers [8]. As a crack propagates through the matrix, various interactions occur between the fibers and the surrounding material. These interactions encompass fiber bridging, de-bonding, pullout, and rupture [6,9,10]. Fiber bridging causes cracks to reduce the stress at the crack location. Meanwhile, the energy absorption during crack propagation is greatly influenced by fiber de-bonding and pullout at the interface. Fiber de-bonding happens when the bond between the fiber and the matrix weakens as the stress at the fiber–matrix interface exceeds the bond strength and results in loss of adhesion. Fiber pullout happens when fibers are partially or completely pulled out from the concrete matrix. Fiber rupture occurs when the fibers themselves break or rupture due to excessive tensile stress. This type of failure typically happens in high-strength fiber-reinforced concretes [11]. Therefore, the strength of the bond between the reinforcing fiber and the surrounding matrix determines the ability of fibers to effectively resist cracks [6].

Up to an optimum fiber content, often expressed as volume fraction, the addition of reinforcing fibers can significantly enhance the mechanical properties and durability of concrete. The volume fraction of fibers measures the volume occupied by the fibers in relation to the total volume of the composite. The extent of improvement in mechanical properties and durability was found to depend on the fiber type, geometry, and content, as well as the binder composition. The average space between fibers decreases as the fiber content increases, thereby limiting the initiation and propagation of cracks within the matrix. Steel fibers are the most investigated in terms of the effect on mechanical properties of concrete [7,12,13,14,15,16,17,18,19,20,21,22,23,24,25,26,27,28,29,30,31]. They generally create a stronger bond with the matrix, leading to higher flexural and tensile performances compared to other types of fibers. Adding steel fibers up to a volume fraction of 1.75% enhanced the post-cracking behavior of alkali-activated GGBS/fly ash concrete, resulting in increased strength, toughness, and ductility [12]. Carbon fibers enhanced the flexural and compressive strengths of concrete, especially after exposure to high temperatures [32]. Adding polyvinyl alcohol (PVA) fibers up to a volume fraction of 1% reduced concrete drying shrinkage by approximately 60% [33]. Polypropylene, basalt, glass, and PVA fibers were also investigated in their various configurations [12,13,28,34,35,36,37,38,39,40,41,42,43]. Some studies have reported the effect of combining different fiber types, or blending different geometries of the same fiber, on concrete properties [12,16]. A hybrid combination of fibers may be formed by blending different types of fiber materials or different geometries/configurations of the same type, merged together at various contents.

Despite the enhancements in mechanical properties and durability, fiber reinforcement can negatively impact flowability and setting time of concretes. The influence can vary significantly based on fiber material type, dosage, and geometry as well as binder type. Rigid fibers form contact networks within fresh concrete composites, increasing their yield stress. When the fiber concentration surpasses a critical level, it causes the formation of fiber chunks or balls and uneven dispersion, impeding the flow of fluid matrices through these networks [18]. The rough surfaces of fibers such as crimped and hooked steel further compromise the workability of fiber-reinforced concretes. The flow of freshly mixed concrete is also affected by the orientation and distribution of fibers. It is important to highlight that an increase in the yield stress of fresh concrete due to incorporation of fibers results in a decline in compressive strength. Therefore, achieving an even distribution of fibers is essential to enhance the overall performance of the concrete [18]. Excessive fiber content introduces voids and defects into the concrete matrix, which affects its performance negatively. Monitoring the workability of fresh concrete is necessary to ensure the attainment of sufficient hardened strength after construction. Ulas [7] reported a sudden decrease in workability when rigid steel fibers content increased to 1.5% within slag-fly ash concrete. High content of steel fibers in concrete pushes aggregates apart, causing slump values to decrease substantially.

**Figure 1:**
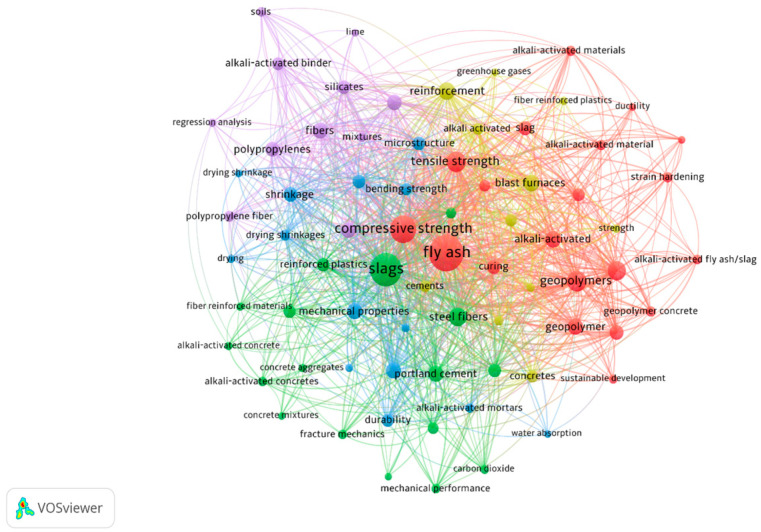
Keywords map of highest relevance to the study using VOS viewer [44].

This article provides a comprehensive overview of the performance of fiber-reinforced concretes in both fresh and hardened states. The review emphasizes key concrete properties that are influenced by reinforcing fibers, such as compressive strength, tensile strength, and durability during service life. In the fresh state, the review focuses on the effect of fibers on concrete flow and workability. The study explores factors influencing the behavior of fiber-reinforced alkali-activated concrete, drawing insights from various literature sources. Figure 1 represents a graphical map of the keywords that are most relevant to this study. Bubble sizes represent the frequency of keywords, while colors display different groups of journals [44]. Most studies analyzed in this article assessed the mechanical properties of fiber-reinforced concrete, while fewer evaluated its durability in challenging environments.

## 2. Properties of Fibers

Fibers that are commonly used to reinforce the binding matrix in concrete are typically made of steel, basalt, carbon, polypropylene, polyvinyl alcohol (PVA), glass, and natural fibers. Each type of fiber features unique characteristics that in turn produce concrete with various properties. Table 1 shows typical mechanical and geometric properties of selected fibers. Many of the fiber types are characterized by substantial tensile strength, which allows them to effectively distribute loads and bridge across cracks.

Fibers can be classified as rigid or flexible depending on their elastic moduli. Rigid fibers are useful in preventing the propagation of cracks before they develop into macrocracks. Flexible fibers, on the other hand, reduce shrinkage cracking and enhance the durability of concrete composites. Steel, carbon, and glass are considered rigid fibers, while polypropylene, PVA, and nylon are considered flexible fibers. The elongation at breakage is another indicator of the ability of fibers to undergo significant deformations before breaking. Fibers with high elongation percentage such as polypropylene and PVA are flexible and more ductile [45,46].

Steel fibers are commonly used in reinforced concrete due to their high tensile strength and modulus of elasticity, which may reach 2.2 GPa and 200 GPa, respectively [47]. The addition of steel fibers to concrete enhances its tensile and flexural strengths, allowing the concrete to withstand strains, absorb energy, and resist cracking even at high temperatures [20,21,48,49]. Steel fibers can be manufactured in various shapes including straight, crimped, and hooked. They also come in a wide range of cross-sectional shapes including circular, rectangular, or square [50]. Hooked steel fibers provide the most effective improvement in the tensile and flexural strengths of concrete compared to straight and crimped types [20,50]. Steel fibers are cost effective in many parts of the world, but their production contributes to significant CO_2_ emissions. Carbon fibers are characterized by tensile strength as high as 3.5 GPa and are therefore used for making fiber-reinforced polymer (FRP) sheets for retrofitting reinforced concrete structures [51]. Carbon fibers have a high melting point of approximately 3650 °C and are considered chemically stable, making them suitable for high-temperature applications and chemically aggressive environments [52].

The incorporation of basalt fibers in concrete enhances its compressive strength and energy absorption capacity. Basalt fibers are cost effective and require a relatively lower amount of energy for their production. Their high tensile strength, low density, and resistance to high temperatures make them suitable for use in various industries [53,54]. Because polypropylene fibers are hydrophobic, they may contribute to the durability of concrete by making structural elements less susceptible to water infiltration [52,54]. However, this hydrophobic nature may weaken their bond with the concrete matrix. Polypropylene fibers have a relatively low modulus of elasticity and a low melting point [52]. These characteristics might limit their use in certain applications related to the construction industry. However, melted polypropylene fibers create channels that not only prevent the spread of fire but also release internal pressure [52]. Polypropylene fibers are highly ductile, with 25% deformability before breakage [45].

Glass fibers offer high tensile strength and high strength-to-weight ratio, which make them suitable for producing lightweight yet strong structural elements. They have been incorporated into different binder compositions for the production of concrete because of their low density, high durability, and good thermal insulation properties [36,55,56,57,58]. However, they reportedly have relatively poorer resistance to moisture and abrasion. Glass fibers are categorized as C-glass, D-glass, R-glass, E-glass, S-glass, and AR-glass [57,58]. Due to its cost-effectiveness, E-glass is the most commonly used type in concrete composites. C-glass fibers are utilized in concretes exposed to acidic environments, while AR-glass is used in concrete with alkali-activated binders due to their ability to resist corrosive effects of alkalis [57]. The production process involves melting broken glass at elevated temperatures, which is then formed into different types of glass fiber products [58].

PVA fibers are hydrophilic and have low density and high resistance to acids and alkalis. Their high elastic moduli and strong adhesion to cementitious matrix make them effective in controlling shrinkage [33,40]. PVA fibers were shown to improve the flexural and compressive strengths of concrete due to the strong bond with the matrix [33,40].

Natural fibers are usually sourced from natural resources such as plants. They are characterized by their low density, availability, and relatively low cost. Plant-based natural fibers, such as flax, jute, cotton, and sisal, were extensively studied [59,60,61,62]. A common significant limitation is their moisture sensitivity, especially plant-based fibers such as cotton and sisal, which can lose strength when exposed to moisture. Furthermore, natural fibers generally exhibit lower tensile strength and stiffness compared to synthetic counterparts, limiting their use in applications requiring high strength or rigidity [63].

The type, content, and geometry of fibers play a critical role in shaping the properties of concrete composites, such as tensile strength, compressive strength, energy absorption capacity, bond-slippage behavior, and crack formation [38,64,65]. Studies have shown that an optimal fiber content exists, depending on fiber type, for concrete mixes to achieve peak fresh properties and hardened concrete to exhibit optimum mechanical strength. With appropriate mixing, water content, binder content, and superplasticizer, a homogeneous concrete mix can be attained up to a particular maximum fiber content, where the significantly higher tensile strength of fibers enhances concrete flexural strength. However, excessive fiber content can lead to poor compaction, increased porosity, and reduced flexural strength [13,32,66]. Random distribution of fibers may decrease the reinforcing efficacy of fibers to 54% compared to perfectly aligned fibers [64]. The bridging mechanism of fibers operates at both micro and macro-levels [15,64]. At the micro-level, they arise from the adhesion and interactions between fibers and the matrix. When a crack approaches a fiber, it requires energy to de-bond the fiber from the matrix. If the interfacial contact zone is inadequate in length or strength, the fiber may de-bond from the matrix. Consequently, cracks can either form, deflect within the material, or branch out into multiple smaller cracks [64].

**Table 1:** Physical properties of different types of fibers.

Fiber Type	Typical Geometric Properties	Density (g/cm^3^)	Elastic Modulus (GPa)	Tensile Strength (MPa)	Elongation (%) at Breakage	Melting Temperature (°C)
Steel	6 mm to 13 mm × 0.75 mm diameter [14]	7.88 [47]	200 [47]	2200 [47]	3 [47]	1200 [67]
Carbon	10 mm × 0.015 mm diameter [68]	1.78 [51]	230 [51]	3500 [51]	1.5 [51]	3650 [69]
Basalt	6 mm × 0.013 to 0.02 mm diameter range [70]	2.63 [71]	79.3 [71]	3000 [71]	3.1 [71]	1100 [72]
Polypropylene	8 mm × 0.033 mm diameter [12]	0.91 [12]	2.8 [45]	500–700 [45]	25 [45]	165 [73]
Glass	12 mm × 0.02 mm diameter [60]	2.5 [56]	82 [56]	2500 [56]	3 [56]	800 [74]
Polyvinyl alcohol (PVA)	8 mm × 0.04 mm diameter [68]	1.3 [46]	42 [46]	1600 [46]	7 [46]	280 [75]
Polyethylene	12 mm × 0.02 mm diameter [76]	0.97 [77]	116 [77]	2900 [77]	2.42 [77]	150 [78]
Jute	35 to 40 mm × 40 to 350 µm diameter range [59]	1.3–1.46 [59]	10–30 [59]	393–773 [59]	1.5–1.8 [59]	140 [79]
Sisal	35 to 40 mm × 50 to 300 µm diameter range [59]	1.45 [59]	38 [59]	600–700 [59]	2–3 [59]	300 [80]

## 3. Effect of Fibers on Fresh Properties of Concrete

Evaluating the fresh properties of concrete is essential to ensure they meet the necessary criteria for proper setting and placement. Fibers can make fresh concrete more challenging to mix, handle, transport, and place, leading to the formation of voids in its hardened state. Factors such as fiber type, length, content, aspect ratio, as well as the binder composition affect workability and setting time. Standardized tests, such as the slump flow [81] T50, and L-Box are used to assess the workability of fiber-reinforced concrete mixtures, while the Vicat needle test is one of the most commonly used methods to assess setting time [82].

### 3.1. Workability/Flowability of Concrete with Added Fibers

Several studies evaluated the fresh properties and rheology of fiber-reinforced mortars and concretes, highlighting the key factors that influence their workability/flowability [12,13,14,15,16,17,18,19,32,34,35,36,61,68,83,84].

#### 3.1.1. Effect of Fiber Type and Content on Workability of Fiber-Reinforced Concrete/Mortar

The workability of alkali-activated concretes is significantly influenced by the fiber content, particularly at higher amounts. The interactions between the fibers and the concrete matrix may hinder the flow of concrete [12,13,32]. Aisheh [12] reported that the addition of 1% steel fibers causes a reduction in the slump value from 190 mm to 170 mm, followed by further decrease to 130 mm when the fiber volume fraction was increased to 2.25%. The decrease in flow occurs as mixes become stiffer with increase in steel fiber content, especially when it exceeds 1.75% [12]. In the same study, when 0.25% polypropylene fiber volume fraction was combined with 2% steel fiber volume fraction, the slump value dropped further to 120 mm [12]. Similarly, Sukontasukkulet [17] reported a decrease in slump flow diameter of alkali-activated concrete consisting of 80% slag and 20% fly ash binders when the volume fraction of hooked steel fibers was increased from 0.5% to 1%. This reduction was attributed to the increased surface area created by the steel fibers, which requires a larger amount of water to maintain concrete flow [17].

The presence of fibers in alkali-activated fly ash and/or GGBS concrete generally results in high shear resistance to flow and compromised workability [13,35,64,85]. Incorporating polypropylene microfibers in fly ash-based concrete reduces the flow diameter values significantly. Irshidat [35] reported that the initial flow diameter of fly ash-based concrete without fibers decreased from 240 mm to 190 mm as the polypropylene fibers volume fraction was increased incrementally to 0.2%. The decrease in workability is attributed to the high yield stress resulting from the interaction of fibers and the concrete matrix, in addition to the high shear resistance caused by the presence of fibers [64,85]. Alkali-activated concrete with 70% fly ash and 30% slag binders also experienced a consistent decrease in flow as the volume fraction of polypropylene fibers was increased incrementally from 0.5% to 2.5% [13]. This decrease in workability is attributed to the increased shear resistance to flow, specifically at higher fiber volume fractions [13].

**Figure 2:**
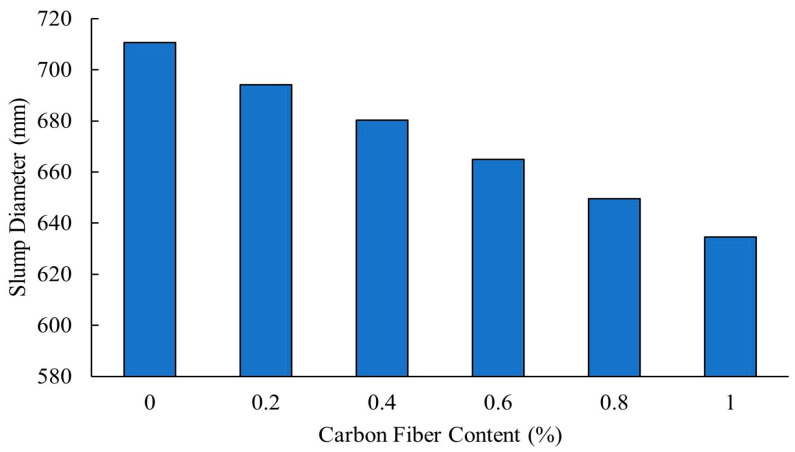
Effect of carbon fiber content on the slump diameter of self-compacting concrete [32].

Khan [32] reported that a carbon fiber content of 0.8% or higher reduced workability of self-compacting concrete due to the increased interactions between fibers, which hindered the flow and influenced ease of placement. Figure 2 shows that as the carbon fibers content was increased incrementally from 0% to 1%, the slump flow of self-compacting concrete decreased consistently. According to the European Federation for Specialist Construction Chemicals and Concrete Systems (EFNARC) standard, the required slump value for concrete is around 650 mm.

Despite the reduction in workability of concrete with fibers, it is possible to design fiber-reinforced self-compacting geopolymer concrete that satisfies the T50, L-box, or other slump flow goals. EFNARC indicates a T50 slump flow time of 2–5 s for acceptable flow of self-compacting geopolymer. Self-consolidating concrete with T50 slump flow time below 2 s is deemed too fluid and may be susceptible to segregation and decreased pumpability or may apply high pressure on formwork. T50 time greater than 5 s is indicative of stiff and non-workable concrete, leading to poorer hardened product with voids. Concrete with long T50 time is also susceptible to segregation and pumpability issues, similar to concrete with very low T50 time. Sukontasukkul [17] reported that self-compacting concrete with less than 1% hooked-end steel fiber content exhibited a T50 time less than 2 s, which falls below the lower limit of the recommended ENFARC range. A hooked-end steel fiber content of 1.5% resulted in self-consolidating concrete with adequate T50 time within the recommended ENFARC range [17].

**Figure 3:**
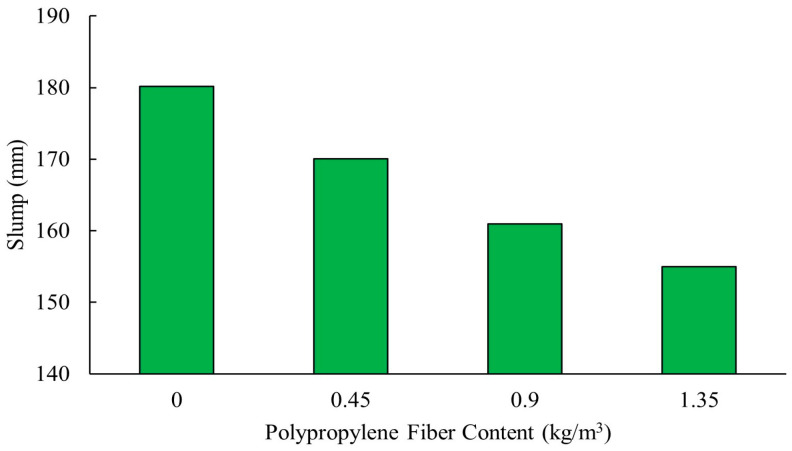
Effect of polypropylene fiber content on the slump diameter of high-performance concrete containing OPC and microsilica [83].

Hydrophobic fibers such as polypropylene tend to impede the flow of the mixes due to their resistance to water, while hydrophilic fibers, such as basalt, interact more favorably with the binder. Figure 3 shows that the slump of high-performance concrete mixes decreased from 180 mm when no fibers were added to 155 mm when the polypropylene fiber content was increased to 1.35 kg/m^3^ [83]. This is because polypropylene fibers have high water absorption characteristics, leading to decreased workability [86]. It is important to note that a steel fiber content of 2.5% caused more significant decrease in slump compared to polypropylene when added to alkali-activated concrete with 70% fly ash and 30% GGBS binders [13].

**Figure 4:**
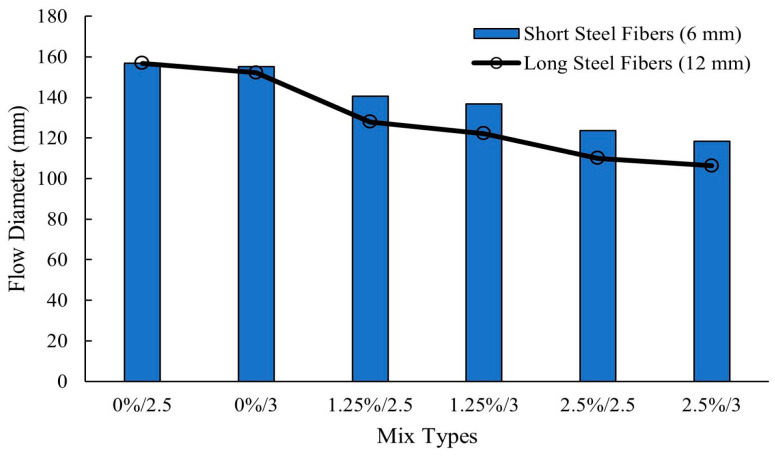
Effect of steel fiber content, fiber length, and activator ratio on the flow diameter of slag-based concrete (0%/2.5 = fiber content/Na_2_SiO_3_/NaOH) [14].

At high volume fractions, long steel fibers have a more significant adverse effect on workability of alkali-activated fly ash/GGBS binders compared to short steel fibers [14,15]. Figure 4 shows that slag-based concrete without fibers experienced the highest workability, which decreased as fiber volume fraction was increased to 1.25%, then to 2.5%. When the alkaline activator ratio Na_2_SiO_3_/NaOH was increased from 2.5 to 3, concrete slump decreased regardless of steel fiber length (6 mm or 12 mm) [14]. Although both long and short steel fibers hindered concrete slump, long fibers caused more pronounced fiber clustering and non-uniform distribution, which led to more pronounced decrease in slump, as shown in Figure 4 [14]. In studies where 20% silica fume was combined with 80% slag, long steel fibers (13 mm) also caused higher reduction in flow diameter compared to short fibers (6 mm) [15].

Bhutta [68] reported that steel microfibers content of 0.5% to 1.0% had the least adverse effect on the flowability of mortar with fly ash binder, requiring little or no vibration, compared to polyvinyl alcohol, polypropylene, polyester, and carbon fibers, which all caused various reductions in mortar flow values. This reduction in workability was accompanied by air entrapment and decreased compressive strength, regardless of the fiber amount or curing regime [68]. Similar to other fibers, the addition of polyolefin fibers caused reduction in flowability of concrete. Zaroudi [61] reported that increasing the volume fraction of polyolefin fibers from 0.25% to 1.25% decreased the workability of OPC-Natural zeolites concrete. When polyolefin fiber content exceeds 1%, the reduction in flow diameter and passing ability of concrete become significant.

#### 3.1.2. Effect of Alkaline Activator Concentration and Type on Workability of Fiber-Reinforced Concrete/Mortar

The alkaline activator solution ratio, Na_2_SiO_3_/NaOH, influences the workability of fiber-reinforced concretes, but its impact is less significant compared to fiber type, dosage, and geometry. Higher Na_2_SiO_3_/NaOH ratios result in greater reduction in workability compared to lower ratios [14]. One reason is that higher concentrations of Na_2_SiO_3_ make the concrete matrix stickier and more viscous. Additionally, higher concentrations of NaOH accelerate the setting time of concrete, reducing its workability [14].

The decrease in flow or workability due to the introduction of reinforcing fibers may remain within acceptance criteria of codes and standard as long as the fiber dosage does not exceed certain thresholds. For concrete with slag-fly ash binders, Prasad [18] reported that the optimal fiber dosages that met the concrete slump criteria according to IS 119-2004 were 0.3% for basalt fibers, 0.55% for crimped steel fibers and 0.1% for polypropylene fiber [18]. For each fiber type and dosage, workability of concrete decreased as the NaOH activator concentration increased from 8 M to 10 M. Sukontasukkul [17] also confirmed that higher NaOH concentrations reduce the T50 time of self-compacting geopolymer mixes.

#### 3.1.3. Effect of Binder Composition on Fiber-Reinforced Concrete/Mortar

The composition of the binder significantly affects the workability of fiber-reinforced alkali-activated concretes. Workability improved with higher fly ash content but was compromised with higher silica fume content [34,36,84]. Karahan & Atis [34] reported that increasing the volume fraction of polypropylene fibers from 0.05% to 0.2% decreases the flow of OPC-based concrete, while replacing 15% to 20% of OPC with fly ash increases the slump of concrete for each of the polypropylene fiber dosages studied. Incorporating mineral admixtures such as fly ash, limestone filler, and ground granulated blast furnace slag may increase the slump of the concrete and reduce the reliance on chemical admixtures [36].

Glass fiber also decreases the slump flow of self-consolidating concrete (SCC) with alkali-activated fly ash and nanosilica binders. Güneyisi [36] reported that increasing the glass fiber content from 0% to 1.5% led to a consistent decrease in concrete flowability. Similar concrete slump flow reduction was observed as the nanosilica content was increased from 0% to 4% of the alkali-activated fly ash binder. This can be attributed to the increased cohesiveness and viscosity of the SCC mixtures due to the presence of nanosilica and glass fibers [36]. Liu [20] reported that ultra-high-performance geopolymer concrete (UHRGC) exhibited a flow of 260 mm without fiber but decreased by 1.2%, 5.4%, and 6.9% as 13 mm corrugated steel fiber dosage was increased to 1%, 2%, and 3%, respectively. This decrease in flowability was attributed to the random distribution of fibers that formed a skeletal structure within the concrete matrix that hindered its flow [20]. Higher steel fiber content enhances their cohesion with the concrete matrix and impedes the flow.

**Figure 5:**
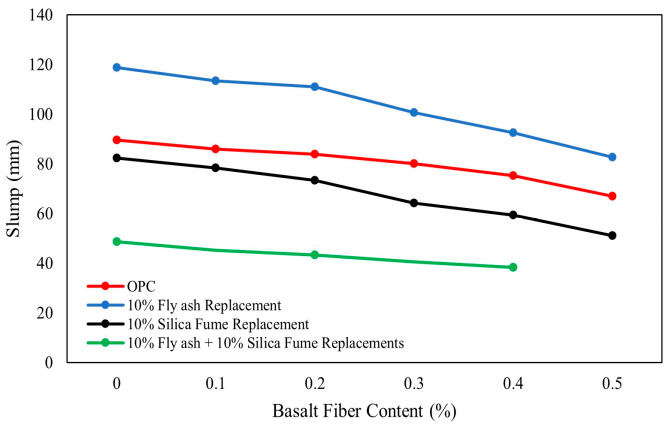
Slump flow values of concretes with fibers across basalt volume fraction ranging from 0% to 0.5% [84].

When basalt fibers dosage of 0.1% to 0.5% was added to OPC-based concrete, or OPC was partially replaced with up to 10% silica fume, a reduction in concrete slump was observed as shown in Figure 5 [84]. The negative impact of silica fume on slump is attributed to its finer particle size and high reactivity [87]. However, replacing 10% of OPC with fly ash increased slump flow from 90 mm for concrete without fly ash to 120 mm with fly ash [84]. This improvement can be attributed to the spherical shape and smooth surface of fly ash particles, which reduces friction and enhances flowability [88].

#### 3.1.4. Effect of Aggregates on Workability/Flowability of Fiber-Reinforced Concrete/Mortar

The gradation of coarse aggregates also influences workability and flow of fiber reinforced concrete. Ramkumar [19] reported higher workability in self-compacting concrete when the amount of small-size aggregates was increased and the reinforcing steel fibers volume fraction ranged from 0.1% to 0.5%. When the amount of smaller-size aggregates is large, concrete with binder consisting of a combination of OPC, GGBS, and microsilica exhibited enhanced workability irrespective to the type and proportion of fibers incorporated, compared to the binder consisting of OPC, Fly ash, and microsilica [19]. Therefore, aggregate size may determine workability of fiber-reinforced concrete as much as the addition of fly ash does. For optimum fresh properties of self-consolidating concrete, Ramkumar and Rajkumar [19] recommended the use of a combination of hooked-end and micro-steel fibers with volume fraction in the range of 0.5% to 0.75%. Beyond the optimum steel fiber content, fiber blockage and friction decrease flowability and restrict spreading of the mix.

#### 3.1.5. Effect of w/b Ratio on Workability/Flowability of Fiber-Reinforced Concrete/Mortar

The w/b and liquid-to-binder ratios significantly influence the flowability of fiber-reinforced concrete [17,61]. Increasing the liquid content in the composite enhances its flow characteristics but may lead to segregation. In fiber-reinforced alkali-activated concrete containing 80% fly ash and 20% slag, water-to-binder ratio of 0.45 provided excellent concrete flowability and stability [17]. Similarly, Zaroudi [61] reported that increasing the water-to-binder ratio from 0.33 to 0.38 in self-compacting concrete results in a 29% reduction in T50 time.

**Figure 6:**
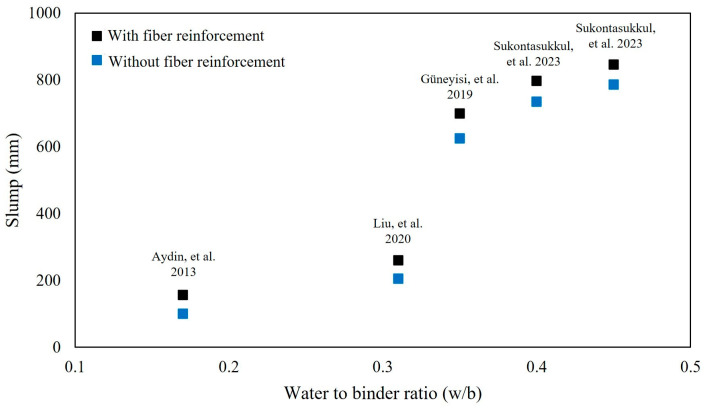
Slump values of concretes with and without reinforced fibers [15,17,20,36].

Data from several studies that evaluated the workability of fiber-reinforced concrete are displayed in Figure 6. As anticipated, slump increases with water-to-binder ratio whether or not fibers were used to reinforce concrete. At all water-to-binder ratios, the addition of fibers to concrete decreases the slump regardless of binder type and fiber dosage.

Table 2 summarizes research findings on the impact of fibers on the workability/flowability of concrete. In general, the addition of fibers reduces the workability of conventional or alkali-activated concrete. The extent of reduction depends on fiber type, fiber content, binder composition, activator type in the case of geopolymers, and liquid-to-binder ratio.

## 4. Mechanical Properties of Fiber-Reinforced Concrete

Reinforcing fibers that are incorporated during mixing affect certain mechanical properties including but not limited to tensile strength, modulus of elasticity, and compressive strength. The most important mechanical properties influenced by the presence of reinforcing fibers are splitting tensile strength and flexural strength. The following subsections discuss the findings of selected literature on the effect of reinforcing fibers on mechanical properties.

### 4.1. Modulus of Elasticity of Fiber-Reinforced Concrete

Reinforcing fibers affect the modulus of elasticity of alkali-activated more than OPC-based concretes, primarily due to the more brittle behavior and lower elastic moduli of alkali-activated concrete [89]. The modulus of elasticity of alkali-activated concretes is highly influenced by the stiffness of fibers [50]. Steel and carbon fibers have relatively higher elastic moduli compared to polypropylene fibers, as discussed in an earlier section and shown in Table 1.

#### Effect of Fiber Type and Content on Elastic Modulus of Concrete

Several studies reported a decrease in the elastic modulus of concrete with reinforced polypropylene fibers [34,35]. Irshidat [35] reported a decrease in the elastic modulus of concrete with fly ash binder from 9.87 GPa without polypropylene fibers to 6.56 GPa when the fiber content was 0.2%. However, at a smaller dosage of 0.05% polypropylene fiber, the elastic modulus of fly ash-based concrete increased in contrast with beams without fibers then decreased when the fiber content was incremented to 0.1% or higher [34].

Steel has a relatively higher modulus of elasticity compared to many other types of fibers, therefore its addition to concrete increases the modulus of elasticity. Yuan et al. [22] reported an elastic modulus of concrete with alkali-activated GGBS/fly ash concrete of 25.6 GPa. The alkali-activated concrete modulus increased to 30.6 GPa when 1.4% corrugated steel fibers was added to the mix [22]. Similarly, Liu [20] reported an increase in the elastic modulus of concrete with alkali-activated GGBS/fly ash binder from 27.6 GPa (steel fiber content = 1%) to 31.5 GPa when the steel fiber content was increased to 3%. Hammad [90] observed a 6.9% increase in the elastic modulus when 1.5% steel fibers was added to slag-based concrete, which is also attributed to the high stiffness of steel fibers and their strong bond with the concrete matrix [23].

In general, the elastic modulus is influenced by the compactness of concrete [24]. Depending on fiber content, fiber type, and mixing method, adding fibers into concrete may enhance its compaction, reducing the occurrence of air voids and mitigating microcracks [24]. Studies by Yang et al. [66] demonstrated that adding basalt, polypropylene, and steel fibers to geopolymer concrete increases the modulus of elasticity. The incorporation of 1% polypropylene fibers or a similar amount of basalt fibers increased the elastic modulus from 27.4 GPa without fibers to 32 and 35 GPa with fibers, respectively. On the other hand, adding 1% steel fibers led to a more significant increase in the modulus from 27.4 GPa to 55 GPa [66]. Similarly, the inclusion of PVA fiber into geopolymer mortar resulted in an increase in the elastic modulus, ranging from 20% to 130%, compared to bench mortar [38]. It is to be noted that the increase in elastic modulus of concrete with increase in the content of reinforcing fibers occurs up to an optimum fiber content, or a threshold, beyond which the elastic modulus decreases. The optimum fiber content depends on several factors including the material and geometry of the fiber [38,66].

### 4.2. Compressive Strength of Fiber-Reinforced Concrete

Pore structure, micro-crack distribution within the concrete, as well as the test boundary conditions influence the compressive behavior of alkali-activated concrete [50,64]. ASTM C39 [91] is one of the international standards used to determine the compressive strength of concrete samples. Fiber-reinforced composites experience a failure pattern where the matrix fails before the fibers do. This allows the fibers to serve as bridges for the propagating cracks through a mechanism that improves the overall performance.

#### 4.2.1. Effect of Fiber Type and Content on Compressive Strength

Fibers with high elastic moduli are reportedly effective in distributing internal compressive forces, preventing stress concentration and the formation of local fractures [64]. Yuan [22] reported an increase in compressive strength of alkali-activated GGBS/fly ash concrete from 37 MPa to 70.8 MPa when corrugated steel fibers were added at a volume fraction of 1.4%. Alkali-activated GGBS/silica fume concrete containing 2.25% steel fibers exhibited a compressive strength of 162 MPa at the age of 28 days, while a combination consisting of 2% steel and 0.25% polypropylene fibers produced concrete with 154 MPa [12]. Similarly, Hammad et al. [90] reported that the inclusion of 1.5% steel fibers in slag-based concrete enhanced the compressive strength by 10.55%, while the addition of 1.5% polypropylene fibers enhanced the strength by 9.11%. The relatively better performance of steel fibers in development of concrete strength is attributed to the stronger bond with the mortar matrix as discussed in earlier sections of this article.

The inclusion of polypropylene fiber in slag-based composite had a more significant impact on its compressive strength and physical properties compared to adding steel fibers [90]. Microscopic analysis of specimens containing polypropylene fiber revealed a strong bond between the aggregate and mortar. The microstructure of steel fiber-reinforced slag-based composites, in contrast, indicated less cohesion between the fiber and the mortar matrix. Samples incorporating polypropylene fibers exhibited a smoother continuity between the mortar matrix and the fibers [90]. This demonstrates improved cohesion compared to the interlocking relationship observed between steel fiber and the mortar matrix in composites containing polypropylene fiber [90].

The porosity of alkali-activated concretes increases when the fibers content exceeds an optimum content [40,85,92]. Increasing the content of basalt fibers to an optimum value of 2% led to a 27.61% increase in compressive strength of concrete with alkali-activated fly ash, GGBS, and rice husk ash binders [70]. Similarly, Zhang [40] reported an increase in compressive strength of geopolymer concrete when PVA fiber content was increased from 0.2% to an optimum value of 0.8%. The percentage increase in compressive strength was lower when PVA content exceeded 0.8%. The decrease in percentile improvement in compressive strength when fiber content exceeds the optimum value was attributed to uneven dispersion and agglomeration of fibers within the geopolymer mortar, resulting in an increase in porosity [40].

Adding 1.75% polyethylene fiber to slag-based concrete led to an increase in the compressive strength, reaching a value of 54.8 MPa [93]. Similarly, Xu [39] reported an increase in compressive strength with the addition of 2% PVA fibers to fly ash-based concrete [39]. At volume fractions up to 0.2%, polypropylene fibers have limited effect on the compressive strength or elastic modulus of OPC-based concrete [34]. In the same mixes, however, replacing 20% of OPC with fly ash resulted in a decrease in compressive strength even with 0.2% polypropylene fibers included in the mix. This is attributed to the slow pozzolanic reactivity of fly ash at early curing ages [94].

**Figure 7:**
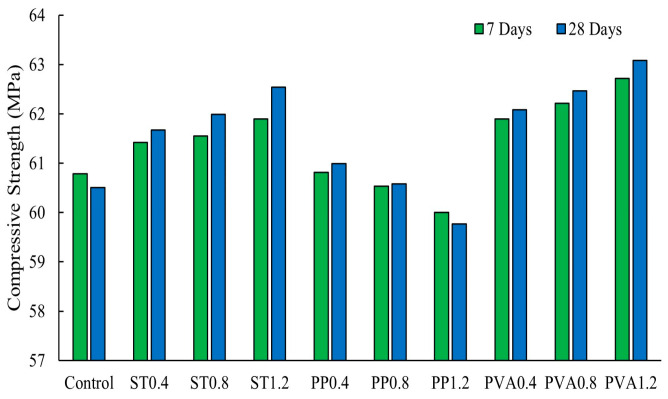
Compressive strength of fiber-reinforced alkali-activated fly ash/slag concretes (ST0.4 = steel fiber content of 0.4%, PP0.4 = polypropylene fiber content of 0.4%, PVA0.4 = PVA fiber content of 0.4%) [33].

Yang et al. [66] reported that adding up to 0.2% basalt, 0.8% propylene fibers, or 1% steel hooked-end fibers increased compressive strength of fly ash-based geopolymer concrete. Concrete with 1% steel fibers exhibited the highest compressive strength of 73 MPa amongst the three types of fibers. This improvement is attributed to the stronger bond between steel fibers and the matrix in comparison to basalt and polypropylene fibers [13,66]. Similarly, Bellum [13] found that steel fiber-reinforced geopolymer composites showed higher compressive strength values compared to composites with propylene fibers at 7 and 28 days, with the highest 28-day compressive strength of 61.5 MPa exhibited by the composite containing 2.5% steel fibers. However, it was observed that increasing the polypropylene fiber content beyond an optimum of 2% volume fraction led to de-bonding between the geopolymer paste and fibers, resulting in a decrease in the flexural strength [13]. Mo [25] reported that adding 0.5% hooked-end steel fibers to fly ash-based concrete led to an increase in compressive strength. In alkali-activated concrete with 70% fly ash and 30% slag binders, the optimum fiber contents for the highest increase in compressive strength were 0.2% polypropylene, 0.4% basalt, and 0.4% steel [26]. Figure 7 shows that the addition of polypropylene, steel, or PVA fibers in the range of 0.4% to 1.2% enhanced compressive strength growth rates at both 7 and 28 days [33]. Concrete containing polypropylene fibers exhibited a decrease in compressive strength as the fiber content increased from 0.4% to 1.2%, while steel and PVA experienced an increase for the same range of fiber contents [33]. However, the enhancement in the 28-day compressive strengths was relatively small.

Khan [32] reported that incorporating up to 0.6% carbon fiber into the concrete led to increased compressive strength to a maximum of 45.34 MP after 28 days of curing. However, increasing the amount of carbon fiber beyond 0.6% resulted in lower gain in compressive strength [32]. Ramkumar and Rajkumar [19] found that the combination of hooked steel and steel microfibers enhanced compressive strength, compared to mixes when the two fiber geometries were added separately to the mixes.

**Figure 8:**
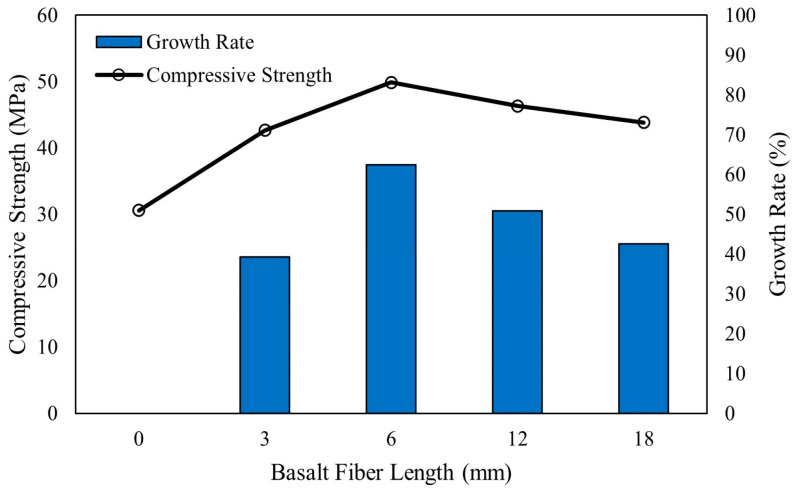
Compressive strength of basalt fiber-reinforced fly ash-based concretes [37].

Fiber length affects the compressive strength of alkali-activated concrete composites, with long fibers causing a reduction in compressive strength [14,37,95]. Compressive strength of geopolymer concrete reinforced with longer carbon fibers was reportedly lower than shorter fibers [95]. Farhan [14] reported that alkali-activated concrete reinforced with 6 mm long steel fibers developed higher 28-day compressive strength compared to 12 mm long steel fibers. Figure 8 shows the effect of the length of basalt fibers on compressive strength development of concrete with alkali-activated fly ash binder [37]. The use of basalt fibers of lengths from 3 mm to 18 mm increases the concrete compressive strength compared to alkali-activated fly ash-based concrete without fibers. The optimum basalt fiber length is 6 mm, as lower strength is developed when fiber lengths were 12 mm and 18 mm. This is because fibers longer than 6 mm cause matrix heterogeneity, which leads to poor bond strength [37].

The addition of natural fibers alongside using industrial byproduct binders in concrete reduces CO_2_ significantly. Wongsa et al. [60] reported that the inclusion of natural fibers led to slight enhancement in the compressive strengths of class C fly ash-based concretes. At a fiber content of 0.05%, the compressive strength of concrete reinforced with sisal and coconut reached 34.7 MPa and 32.4 MPa, respectively, exceeding the compressive strength of the control mix, which was 30.4 MPa. When the added natural fiber exceeded an optimum content of 0.05%, the concrete compressive strength decreased for all fiber types. Higher fiber contents led to uneven dispersion of fibers and increased the voids in the composite [60].

#### 4.2.2. Effect of Curing Conditions on Compressive Strength of Fiber-Reinforced Concrete/Mortar

Fiber-reinforced composites cured in water demonstrate better compressive strength development compared to those cured in sealed conditions. This improvement is attributed to the ongoing formation of geopolymerization products facilitated by the presence of water [47,96]. Similarly, thermal curing up to a certain temperature promotes faster geopolymerization and reduces the potential for shrinkage cracks. Sathandam [56] reported that adding 0.3% glass fiber to thermally cured fly ash-based concrete increased the compressive strength [56]. Fly ash-based concrete typically requires heat curing to accelerate the geopolymerization reaction. Ohno and Li [97] reported that heat curing also enhances the compressive strength of fiber-reinforced concretes made with fly ash. Fly ash-based concrete reinforced with PVA and heat cured for 8 h exhibited higher compressive strength values than those cured for 4 h [97].

The addition of polypropylene fibers in the range of 0.45 kg/m^3^ to 1.35 kg/m^3^ had a minimal impact on the compressive strength of ultra-high-performance geopolymer concrete after 7 days of curing. However, after 28 days of curing, concrete reinforced with polypropylene fibers exhibited higher compressive strengths than the concrete without fibers [83]. Abdollahnejad [47] reported that higher fiber content consistently resulted in higher concrete compressive strength, irrespective of the curing age and fiber type. The most significant increase, exceeding 50%, was observed in concrete containing 1% PVA or steel fibers. In contrast, polypropylene fibers showed the lowest compressive strength improvement, primarily due to their weaker bond with the matrix [47].

Zhang et al. [40] reported that geopolymer mortar reinforced with PVA fibers outperformed mortar without fibers in terms of compressive strength at temperatures up to 200 °C. The addition of 0.8% PVA fiber led to a significant increase in compressive strength, surpassing that of the mortar without fibers by 35.6% at 25 °C and 50.5% at 200 °C. However, as temperature exceeded 200 °C, a significant decline in compressive strength was observed in the geopolymer mortar [40].

#### 4.2.3. Effect of w/b Ratio on Compressive Strength of Fiber-Reinforced Concrete/Mortar

Lower w/b ratios promote better bonding between the fibers and the concrete matrix [98]. As indicated earlier in this article, increased water content may be necessary to enhance flow/workability of fiber-reinforced concrete/mortar. In PVA fiber-reinforced slag-based concrete, the w/b ratio significantly influences the compressive strength. A w/b ratio of 0.34 resulted in the highest compressive strength of 30.6 MPa, exceeding the strengths achieved at higher w/b ratios [98].

#### 4.2.4. Effect of Alkaline Activator on Compressive Strength of Fiber-Reinforced Concrete/Mortar

The molarity of the activator affects the compressive behavior of fiber-reinforced alkali-activated concrete. Prasad et al. [18] reported that fiber-reinforced alkali-activated fly ash/slag concrete activated with 10 M NaOH exhibited higher compressive strength than concrete with binder activated with 8 M NaOH solution at the ages of 7 and 28 days. This effect can be attributed to the higher alkalinity and reactivity of the 10 M NaOH solution, which facilitated the activation of fly ash and slag particles, leading to the formation of a denser and stronger concrete matrix [99].

**Figure 9:**
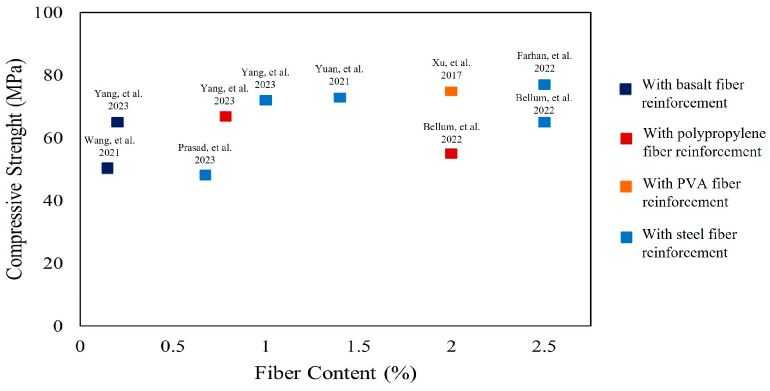
Compressive strength of alkali-activated GGBS/fly ash concretes and mortars reinforced with different fibers [13,14,18,22,37,39,66].

Figure 9 shows the effect of fiber content on the compressive strength of concretes and mortars with alkali-activated GGBS and/or fly ash binders, reinforced with basalt, polypropylene, PVA, and steel fibers, independently. Each data point corresponds to a compressive strength value for a specific mix. It may generally be noted that increase in fiber content yields higher compressive strength values at the same fiber type, depending on the binder composition [13,14,18,22,37,64]. Additionally, steel fibers yield higher compressive strength compared to polypropylene for the same binder composition [13,64]. Including fibers within the concrete mix may enhance compressive strength; however, this enhancement is limited compared to improvement in flexural strength.

Table 3 summarizes research findings on the impact of fibers on the elastic modulus and compressive strength of fiber-reinforced composites.

### 4.3. Splitting Tensile Strength of Fiber-Reinforced Concrete

The tensile strength of alkali-activated concretes is significantly improved with the addition of reinforcing fibers. In general, when alkali-activated concrete without fibers is subjected to tensile forces, it experiences multiple splitting tensile failure surfaces due to its brittle behavior. The splitting tensile strength is usually tested on cylindrical concrete specimens based on commonly accepted standards, such as ASTM C496 [105]. Studies have shown that adding an appropriate amount of fibers will prevent the formation and expansion of internal cracks in concrete [22,37,70]. This is because fibers act as reinforcement within the concrete matrix, effectively distributing tensile stresses that arise during loading or as concrete experiences shrinkage.

The failure patterns of steel fiber-reinforced alkali-activated concretes differ significantly from those without steel fibers. Under tensile stresses, concrete without steel fibers is typically brittle and exhibits multiple splitting tensile failure surfaces, while concrete with steel fibers primarily fails in shear and shows improved ductility [22].

#### 4.3.1. Effect of Fiber Type and Content on Splitting Tensile Strength of Concrete

Fibers with high tensile strength contribute significantly to enhancing tensile strength of alkali-activated concrete. However, the incorporation of fibers with lower tensile strength, such as polypropylene fiber, has a limited effect on concrete tensile strength. Liu [20] reported that increasing steel fiber content from 1% to 2% results in a 14.5% enhancement in concrete tensile strength, while further increase in fiber content to 3% leads to a substantial 42.1% increase in splitting tensile strength. When added to concrete, basalt fibers enhance its energy absorption capacity and reduce cracking [37]. Zhang [28] reported a 27.4% increase in tensile strength compared to unreinforced specimens when concrete was reinforced with a combination of basalt and steel fibers. The energy absorption experienced by fiber-reinforced composites is due to the various types of interactions between fibers and the matrix. As a crack propagates through the matrix, fibers may experience bridging, de-bonding, pullout, and rupture [6].

Fibers are effective in enhancing the tensile strength up to an optimum dosage that depends on the type of fiber. Incorporating fiber in excess of the optimum dosage can introduce numerous voids and defects into the concrete matrix. Yang [66] reported 0.2% basalt fibers as an optimum content that yields the maximum splitting tensile strength. This is consistent with the optimum basalt fiber content reported by other researchers [37]. Similarly, an optimum fiber content for maximum splitting tensile strength of 0.8% was reported for polypropylene, and 1% for hooked steel fibers.

The length of the fibers also influences the tensile strength of fiber-reinforced concretes. Keeping in mind the concept of optimum values, longer fibers tend to provide a greater enhancement in tensile and flexural strength than shorter fibers [14,106]. Longer fibers offer a greater contact surface area with the matrix, allowing them to form strong interfacial bonds leading to effective transfer of tensile stresses in the flexural zone [14]. Short fibers do not provide as much contact and bond surface area due to their smaller length-to-diameter ratio, which results in a relatively lower enhancement in flexural strength [14,38]. A significant increase in tensile strength of slag-based concrete was reported when the mix was supplemented with 2.5% long steel fibers [14]. Wang et al. [37] reported that incorporating basalt fibers up to a length of 6 mm led to improvement in splitting tensile strength compared to a lower value for concrete without basalt fibers. However, using basalt fibers longer than 6 mm may cause matrix heterogeneity due to clustering of fibers and formation of voids and gaps between fibers and matrix, which subsequently reduces bond strength [37].

Adding properly selected combinations of short and long fibers may lead to improvement in splitting tensile strength of alkali-activated concrete [12]. When using a combination of different lengths of fibers in concrete mixes, two main mechanisms contribute to crack resistance. Shorter fibers can effectively bridge tiny fractures in the concrete, creating better connections between micro-fractures and minimizing the formation of larger cracks with wider widths. Longer fibers may hinder the rotation of shorter fibers and work in synergy with them, enhancing the overall performance of the concrete by providing additional reinforcement [12]. Zhang et al. [28] reported that using a combination of basalt and steel fibers in concrete results in a 27.4% increase in tensile strength when compared to unreinforced samples [28].

From a failure mechanism perspective, steel fibers create small fractures in the concrete during fiber pull-out, whereas polypropylene fibers are more prone to breaking [29]. However, the combination of polypropylene and steel fibers results in an overall enhancement of the strength and performance of concrete with alkali-activated binder consisting of 70% GGBS and 30%. Combining 0.25% polypropylene fibers with 2% steel fiber enhanced the fiber ability to bridge across fractures and led to higher tensile strength compared to the alkali-activated concrete without fibers [12].

Xu et al. [39] reported that increasing PVA fiber content from 0% to 2% improves compressive, tensile, and flexural strengths of fly ash-based concrete. After 28 days of curing, fly ash-based concrete with 2.0% PVA fibers showed an improvement of 70% in compressive strength, 220% in tensile strength, and 270% in flexural strength compared to fly ash-based concrete without fibers [39]. Microscopic analysis revealed the formation of continuous geopolymer gel network, which boosted toughness and compacted the interfacial transition zone. Additionally, film-like substances were observed on PVA fiber surfaces, which enhanced matrix bonding and the performance of geopolymer composite [39]. When steel or macro-polypropylene fibers in a range of 0.3% to 0.6% were added to slag-based concrete, the tensile strength did not significantly increase. Steel fibers yielded slightly higher enhancements in splitting tensile strength than macro-polypropylene fibers. This limited impact of steel fibers on the tensile strength could be attributed to the relatively small fiber content and the smooth surface fiber characteristics, resulting in lower bond strength [30].

#### 4.3.2. Effect of Binder Composition on Splitting Tensile Strength of Fiber-Reinforced Concrete

The inclusion of steel fibers in fly ash-based concrete was found to improve its mechanical properties [25]. The increase in compressive strength was relatively lower compared to splitting and flexural tensile strengths [25]. This is because the fibers do not actively contribute to load sharing in the compression direction since they do not undergo stretching and straining under compression. Instead, they enhance concrete tensile strength by distributing tensile stresses more uniformly and enhancing load-sharing capability. This allows the concrete to absorb more energy and undergo significant plastic deformation. Hung [25] indicated that the addition of 0.5% hooked-end steel fibers in fly ash-based concrete with lightweight aggregate resulted in an increase in post-peak behavior, fracture toughness, and a substantial 57% increase in splitting tensile strength. This enhancement is attributed to the crack-bridging ability of the fibers, leading to delayed propagation of cracks. Hammad [90] indicated that incorporating fibers in slag-based concrete yielded significant enhancements in tensile strength. Slag-based concrete containing polypropylene fiber exhibited an improvement of 19.28% in tensile strength, while those with steel fiber showed an increase of 26.81% compared to the unreinforced composites. These enhancements are attributed to the random and homogeneous orientation of the added fibers, which effectively filled the inter-particles between the aggregate and the mortar matrix. This prevented the extension of microcracks and contributed to the overall strength of the specimens [90].

### 4.4. Flexural Strength of Fiber-Reinforced Concrete

When subjected to tensile stresses, concrete tend to fail in three different modes: opening mode, tearing mode, and sliding mode. The presence of fibers in alkali-activated concretes significantly enhances the flexural strength by providing reinforcement and resistance to applied loads. The flexural strength of fiber-reinforced concrete is assessed using various international standards, such as ASTM C1609 [107]. The test involves subjecting a simply supported beam to third-point loading using a closed-loop, servo-controlled testing system. The test measures the peak load and calculates the corresponding stresses.

Effective positioning of fibers within the hardened concrete matrix enables stress transfer along the reinforcing fibers, while the fibers themselves bear loads in their longitudinal directions [50]. Certain surface treatments or fiber modifications were proposed to control composite bonding and slip hardening behavior. For example, oil treatment can tailor the interface between fibers and the geopolymer matrix [64].

#### 4.4.1. Effect of Fiber Type and Content on Flexural Strength of Concrete

The flexural strength of alkali-activated concretes is influenced by the amount of reinforcing fibers. Lower fiber content can enhance flexural strength by improving the homogeneity of the matrix but may not be sufficiently effective. Conversely, excessive fiber content can lead to a porous and heterogeneous structure, weakening the composite due to stress concentration. Ziada [54] reported that adding up to 1.2% basalt fibers enhanced the flexural strength of fly ash-based concrete by up to 44% through effective transfer of flexural loads and decrease in the number of fractures during flexure test [54].

**Figure 10:**
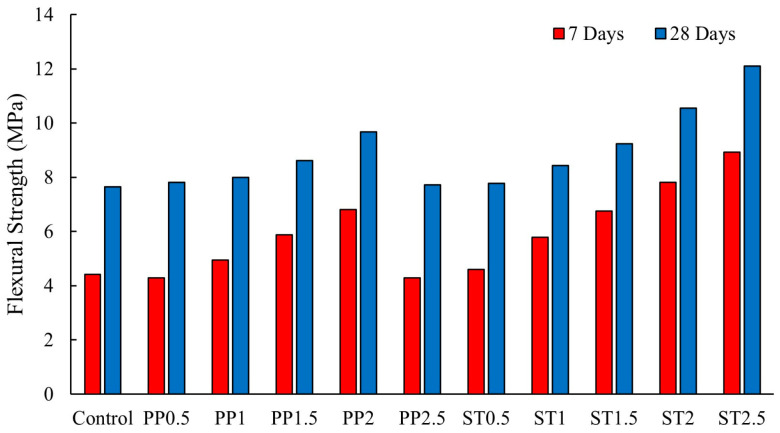
Flexural strength of fly ash-based concretes reinforced with polypropylene and steel fibers (PP0.5 = polypropylene fiber content of 0.5%, ST0.5 = steel fiber content of 0.5%) [13].

When alkali-activated concretes are subjected to bending loads, the tensile stress generated is transformed into shear stress at the fiber–matrix interface. The strength of this interaction can vary depending on the type of fiber used [13]. According to Bellum [13], the addition of 2.5% steel or polypropylene fibers resulted in a maximum enhancement in 28-day flexural strength of 57.79% and 26.36%, respectively. This improvement is attributed to the strong bond between the fibers and the geopolymer matrix, with steel fibers displaying superior bond in comparison to polypropylene fibers [13]. Figure 10 shows that increasing polypropylene fiber content beyond 2% volume fraction leads to de-bonding between the geopolymer paste and fibers, resulting in a decrease in the flexural strength. The polypropylene microfibers proved to be effective in preventing the formation and propagation of microcracks caused by tensile stresses in the lower region of the specimens during the flexural strength test [13]. The presence of fibers plays a significant role in halting the conversion of microscale cracks into macroscale cracks, thereby maintaining the integrity of the specimens [35].

#### 4.4.2. Effect of Curing Conditions and High Temperatures on Flexural Strength of Concrete

Curing conditions impact the flexural behavior of fiber-reinforced composites significantly. Abdollahnejad [47] found that water curing enhances flexural strength development of alkali-activated slag-based concrete. The submersion of samples under water maintains the formation of products such as C-A-S-H gel, which increases both mass and strength over time [96].

Alkali-activated binders made with 50% metakaolin and 50% fly ash and reinforced with 2% short carbon fiber provided higher flexural and compressive strength both at ambient temperature and after exposure to high temperatures compared to the binder without carbon fiber [101]. This improvement is attributed to the reinforcing fibers acting as crack arresters and stress distributors. The flexural and compressive strength of metakaolin/fly ash paste, mortar, and concrete compositions demonstrated a comparable behavior to OPC mixtures with a slight increase after exposure to 100 °C and then decrease in the range of 100–800 °C [101]. He [108] reported that heat treatment of metakaolin concrete within the temperature range of 1100 to 1300 °C had a pronounced impact on the microstructure and mechanical attributes of the composites. At 1100 °C, the composite exhibited substantial enhancement in flexural strength and Young’s modulus compared to its initial state [108]. The composite under an elevated temperature of 1100 °C exhibited a peak flexural strength of 234.2 MPa and a Young’s modulus value of 63.8 GPa. These improvements are due to the formation of a densified and crystallized matrix, along with enhanced bond between the fiber and matrix interface [108]. However, at 1400 °C, mechanical properties deteriorated significantly due to severe carbon fiber degradation, matrix melting, and crystal phase dissolution, resulting in brittle behavior [108].

The flexural strength of both steam and standard-cured ultra-high-performance geopolymer concrete increases as the fiber content increases [65]. A substantial improvement in the ultimate flexural strength of both steam and standard-cured geopolymer concrete occurred with increase in fiber content. When 3% steel fibers was mixed with geopolymer concrete, the flexural strengths at standard curing increased by 434.1%, while the flexural strengths of steam-cured samples increased by 189.4% [65].

High temperatures exceeding 200 °C may cause a significant reduction in flexural and compressive strength of fiber-reinforced composites. Ziada [54] reported that adding 1.2% basalt fibers to fly ash-based mortars at temperatures below 200 °C increased the flexural strength from 10 MPa to 13 MPa. The flexural strength of the composite containing 1.2% basalt fibers and exposed to 200 °C, 400 °C, 600 °C, and 800 °C decreased by 2.27%, 14.02%, 33.71%, and 44.32% compared to samples not exposed to high temperatures. This decrease is attributed to the increased internal pressure at high temperatures, which led to microcracks [54]. Zhang [101] reported a more significant decrease in flexural strength at high temperatures within geopolymer pastes than mortar and concrete. This difference is attributed to the higher fiber content in pastes, making their strength more reliant on the reinforcing function of the fibers. On the other hand, mortar and concrete include aggregates that offer supplementary strength and stability, thereby alleviating the impact of fiber degradation to a certain degree [101].

#### 4.4.3. Effect of Binder Composition on Flexural Strength of Fiber-Reinforced Concrete

The flexural strength enhancement by glass fibers was found to depend on the binder composition [55,56]. Sathandam [56] studied the influence of glass fiber contents ranging from 0.1% to 0.5% on the flexural strength of thermally and naturally cured alkali-activated fly ash-based mortars. Sathandam [56] reported that glass fibers within the range of 0.1% to 0.5% increase the flexural strength for both heat and ambient-cured fly ash-based composites. However, Puertas [55] reported that sodium silicate-activated slag mortars are not affected by the incorporation of glass fibers at fiber content ranges from 0.11% to 1.1%. Therefore, low glass fiber content does not significantly improve flexural strength of concrete with slag binders, unlike fly ash binders [55].

The post-cracking behavior of the material can be significantly improved, resulting in increased strength, toughness, and durability [10,109]. Flexural toughness and post-cracking ductility of fiber-reinforced ladle slag-based concrete were investigated by Natali et al. [109]. Results revealed that the post-cracking behavior is significantly improved when incorporating fibers. Reinforcing fibers extend the non-linear portion of the load–deflection curve, leading to improved energy absorption during fracture [109]. Carbon and PVC fibers produce concrete with higher fracture toughness and ductility. These fibers display an enhanced ability to bridge gaps within the cracked material matrix due to their favorable pull-out mechanism exhibited when fracture occurs [109].

Bernal [27] reported that the modulus of rupture of geopolymer concrete increases with curing age over 7, 28, and 90 days. This is due to the ongoing development of geopolymerization products over time. The addition of steel fibers enhances the post-cracking behavior and load capacity of slag-based concretes. This is due to the increased crack tortuosity caused by the addition of steel fibers [27].

**Figure 11:**
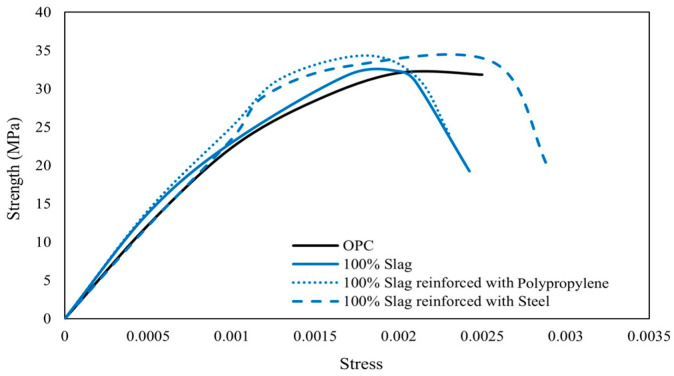
The stress–strain curves for OPC and slag-based concrete reinforced with polypropylene and steel fibers after 28 days [90].

The stress–strain curves shown in Figure 11 reveal distinct flexural behavior between OPC concrete and slag-based concretes. Although all concrete types achieved the same target compressive strength of 35 MPa, their responses to increasing strain differ significantly. The stress–strain curves for slag-based concretes with and without fibers initially exhibit a linear pattern similar to that of OPC concrete. However, when the ultimate strength is reached, slag-based concrete exhibits lower strains compared to the OPC composite [90]. When steel fibers were added to slag-based concrete, the composite experienced high strain values. Therefore, steel fiber-reinforced slag-based concrete offers high toughness and ductility, with the ability to absorb more energy during deformation and resist crack formation and propagation [90]. After reaching the peak compressive strength, the stress in concrete decreases gradually, reflecting the onset of deformation and microcracking.

The load is effectively carried by steel fibers in the post-cracking stage, resulting in improved flexural toughness [65]. The presence of steel fibers influences the pre-cracking behavior, and the initial crack behavior is directly influenced by the steel fiber content. An increase in steel fiber content leads to a higher energy absorption capacity [65]. Liu [65] demonstrated that increasing the steel fiber content enhances the first crack and peak deflection in ultra-high-performance geopolymer concrete. At 1% fiber content, the first crack deflection and peak deflection were 0.066 mm and 0.75 mm, respectively. These values increased to 0.089 mm and 0.85 mm when fiber content increased to 3%, indicating higher deflections with higher fiber content.

The addition of steel fibers to slag-based concretes results in higher flexural strength and better post-peak behavior than fly ash-based or OPC-based concretes [30]. Slag-based concrete demonstrated a post-hardening behavior after cracking within the deflection range of 0.2–3.5 mm. Similarly, the modulus of rupture of concrete increases with the content of steel fibers and concrete curing age. This is attributed to the resulting denser matrix of the composite and the high modulus of elasticity of steel fibers [27].

The bond between fibers and the geopolymer matrix is stronger than fibers with cement matrix. Zhang et al. [110] reported superior bond strength between PVA fiber and geopolymer mortar compared to the bond between PVA fiber and cement mortar. The addition of PVA fibers to geopolymer mortars has a pronounced positive impact on flexural strength [110].

Using steel fibers to reinforce alkali-activated slag/fly ash concrete columns enhanced their axial load-carrying capacities, ductility, and flexural load resistance under concentric and eccentric axial loads. Incorporating hybrid steel fibers resulted in a 14.6% increase in axial load capacity, 146.2% increase in ductility, and 26% improvement in flexural load resistance compared to columns without steel fibers [16]. This is due to the ability of various steel fiber types and sizes to hinder crack formation at both the micro and macro-levels.

Hameed [111] proposed a finite element model for evaluating the compressive and tensile properties of steel fiber-reinforced concrete structures. The model for steel fiber-reinforced concrete structure combined an isotropic elastic-plastic damage model with a sub-model that addresses the viscous damage effects within the steel fibers matrix. This modeling approach captured the response of concrete structures reinforced with steel fibers to various loading conditions and potential damage mechanisms [111]. Buratti [112] developed a design approach for steel fiber-reinforced concrete linings in real practical applications using numerical modeling. Relationships between stress and strain were derived, as well as stress and crack development, through a series of four-point bending tests. The design procedure involved an active hinge and stress–crack tensor relationships, offering a systematic approach to ensure the structural integrity and safety of these linings. Results showed that steel fiber-reinforced concrete linings outperformed plain concrete linings even in unpredictable load situations. Higher steel fiber dosage in reinforced concrete linings led to greater increments of bending, which indicates an increase in the safety margin [112].

#### 4.4.4. Effect of Alkaline Activator on Flexural Strength of Fiber-Reinforced Concrete

**Figure 12:**
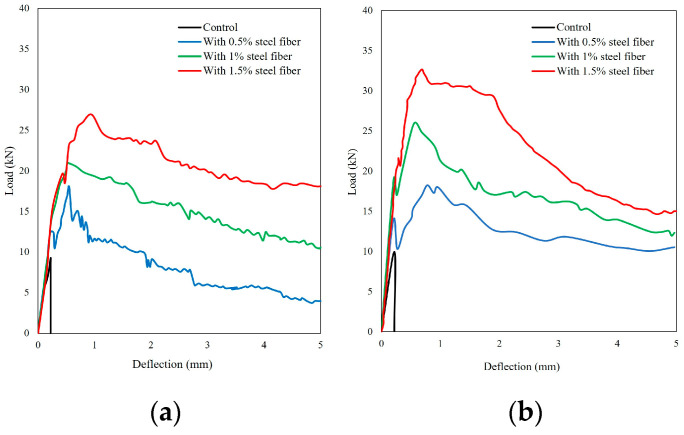
Flexural behavior of self-compacting geopolymer concrete reinforced with steel fibers at NaOH concentrations of (**a**) 8 M and (**b**) 12 M (Control = without fiber reinforcement) [17].

For steel fiber contents varying from 0% to 1.5% volume fractions, the flexural strengths of composites with NaOH concentrations of 8 M and 12 M increased from 2.84 to 5.95 MPa and from 3.10 to 6.54 MPa, respectively. This improvement in flexural strength was primarily attributed to the bridging effect of fibers, which increased the load-carrying capacity of geopolymers [17]. Figure 12 shows the load response of plain fly ash-based concrete, which starts by linear increase in load corresponding to deflection until reaching the peak load, followed by abrupt failure and a drop in load [17]. In contrast, fly ash-based concrete reinforced with steel fibers exhibited a considerably ductile behavior. The load increased linearly with deflection until the peak load, at which cracks developed. However, the load did not experience an abrupt decline; instead, it gradually decreased with deflection due to the bridging effect of fibers across cracks, impeding crack growth [17]. Higher steel fiber contents led to an increase in peak load and ductility that is more significant in concrete with 12 M NaOH solution than 8 M solution [17].

**Figure 13:**
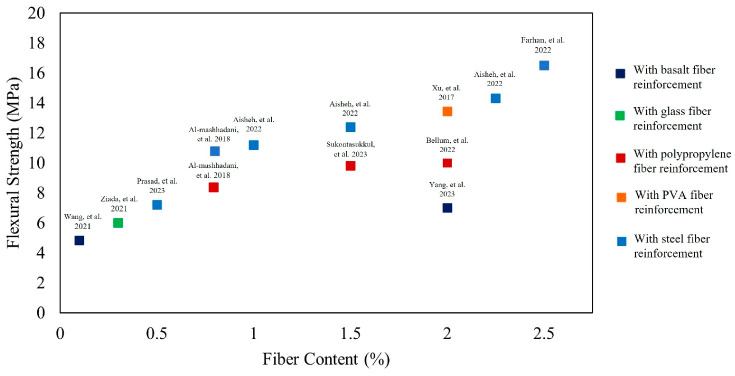
Flexural strength of fiber-reinforced concretes [12,13,14,17,18,33,37,39,54,66].

Figure 13 presents data collected from several experimental studies on the flexural strength of fiber-reinforced concretes. The use of basalt, glass, polypropylene, PVA, and steel fiber reinforcements in cementitious composites results in enhanced flexural strength, which is attributed to the strong bond between the fibers and matrices. It is generally noted that higher fiber content results in higher flexural strength regardless of the fiber type. At the same fiber content, steel fibers produce concrete with higher flexural strengths compared to polypropylene [12,17,33].

Table 4 displays research findings on the impact of fibers on the splitting tensile and flexural strengths of fiber-reinforced composites.

### 4.5. Toughness and Fracture Energy of Fiber-Reinforced Concrete

The addition of fibers enhances the energy required for a crack to propagate into concrete [54]. The toughness factor, an indicator of the toughness of a material, is typically determined from the load–deflection curve using Equation (1) based on ASTM C1609 [107].

(1) $$\sigma =\frac{T_{b}}{\delta_{tb}}\times \frac{L}{bh^{2}}$$

- *δ_tb_* = deflection at 1/150 L.
- *T_b_* = flexural toughness at 1/150 deflection (area under the load–deflection curve).
- *L*, *b*, and *h* = length, width, and height of the sample in (mm), respectively.

#### 4.5.1. Effect of Fiber Type and Content

The addition of steel fibers to the composite positively affects the toughness of fiber-reinforced concrete at room temperature. However, polypropylene fibers were found to have a minimal impact on toughness [106]. Bellum [13] reported that adding steel fibers at a volume fraction of 1.2% increased the flexural toughness by 273.42% compared to the control mix. Polypropylene fibers beyond 2% volume fraction resulted in a decrease in the flexural toughness. This is possibly due to the de-bonding between the geopolymer paste and fibers at high fiber contents [13]. Increasing the steel fiber content enhances the first crack deflection, strength, and peak deflection in ultra-high-performance concrete. At 1% fiber content, the values for first crack deflection, flexural strength, and peak deflection are 0.066 mm, 3.28 MPa, and 0.75 mm, respectively, while at 3% fiber content, these values increase to 0.089 mm, 5.75 MPa, and 0.85 mm, respectively [65]. Moreover, an increase in fiber content leads to a higher energy absorption capacity during the cracking process. In the post-cracking stage, the load is effectively carried by the steel fibers, resulting in improved flexural toughness [65].

Incorporating steel fibers yielded a significant toughness enhancement, with the fiber-reinforced alkali-activated slag and silica fume mortar exhibiting toughness values up to 12,500% higher than the control mortar without fibers. Particularly, longer steel fibers of 12 mm exhibited the highest energy absorption across all dosages [15]. This notable improvement can be attributed to the distinct capability of steel fibers to address cracks at both micro and macro-levels [15]. At the micro-level, these fibers act as inhibitors that effectively prevent crack initiation. Meanwhile, at the macro-level, the steel fibers play a bridging role by spanning cracks and evenly distributing stresses within the material [15]. The combined effect of these micro and macro-scale actions prevents crack propagation and significantly improves the composite’s overall toughness and ductility.

The length of PVA fibers has a notable influence on fly ash-based concrete, but the effect on strength is relatively minor [39]. The enhancement in toughness is more significant in samples containing 12 mm PVA fibers in comparison to those utilizing 8 mm PVA fibers, with a flexural toughness index surpassing that of the control sample by nearly 1300% [39]. The increased toughness of fly ash-based concrete can be attributed to the superior bridging effects and interlocking network provided by the longer fibers, resulting in enhanced resistance to cracking and deformation [39]. Zhang [38] reported that the peak load increases significantly with the increase in the amount of PVA fiber up to an optimum content of 1.0%. The critical effective crack length undergoes a sharp rise after reaching 0.4% PVA fiber content and then remains relatively stable after 0.6%, indicating notable improvements in the mortar’s stability at higher PVA fiber contents. Similarly, fracture toughness of geopolymer mortar demonstrates a sharp increase starting at 0.4% PVA fiber content and continues to grow until the content reaches 1.0% [38]. Notably, the fracture energy determined in this study is approximately tenfold higher than that of geopolymer mortars without fibers [38].

Wang [37] reported that the length of fibers has a significant impact on the fracture energy of basalt fiber-reinforced fly ash-based concrete. Fracture energy of fly ash-based concrete increased with the addition of 3 mm basalt fibers then increased further when the basalt fibers used were 6 mm long. However, 6 mm was an optimum basalt length, as fracture energy decreased when the fiber length was increased to 12 mm and decreased further when fiber length was 18 mm [37].

#### 4.5.2. Effect of Alkaline Activator

Incorporating fibers in concrete structural elements leads to a significant enhancement in fracture energy and an increase in material ductility. Fracture energy of fiber-reinforced concrete beams is highly influenced by fiber type, fiber content, binder type, and concentration of activator solution in the case of alkali-activated binders. Adding 3 kg/m³ polypropylene or basalt fibers to concrete with 12 M NaOH activator exhibited improved fracture energies of 474.832 N-mm and 472.232 N-mm, respectively. In contrast, the same content of glass fibers produced concrete with lower fracture energy of 401.369 N-mm when the NaOH activator concentration was 4 M [114]. For a particular fiber type and when the activator concentration is not a factor, the difference in fracture energy is attributed to the distribution of fibers in the matrix. Scanning electron microscopy (SEM) images revealed a uniform distribution for basalt and polypropylene fibers and some clustering in glass fibers when used to reinforce concrete [114]. Alkali-activated slag-based concrete containing polypropylene fibers outperformed those with basalt and glass fibers in terms of deformation capacity, energy dissipation, and overall mechanical performance [114].

## 5. Durability Properties

Concrete is vulnerable to durability issues in harsh environments. Evaluating the long-term performance and durability of fiber-reinforced concrete structures is critical to ensure their longevity and integrity in harsh environmental conditions. Adding fibers enhances the durability of concrete by enhancing resistance to water absorption, chloride penetration, carbonation, shrinkage, and freeze–thaw damage.

### 5.1. Shrinkage

Shrinkage of concrete typically causes non-uniform volume changes along with tensile stresses. If the tensile strength of concrete is exceeded, the resulting cracks could facilitate the transport of deleterious materials that could not only affect the integrity of concrete but also reach and potentially corrode reinforcing steel bars [120]. Fibers contribute to reducing drying shrinkage by influencing the crack development and propagation within the matrix. These toughening mechanisms, operating at both micro and macro-levels, can affect the formation of extensive cracks that contribute to drying shrinkage. Using high-stiffness fibers was found to prevent crack localization and expand the effective crack path, ultimately reducing the drying shrinkage in fiber-reinforced composites [121].

#### 5.1.1. Effect of Fiber Type and Content on Shrinkage of Concrete

**Figure 14:**
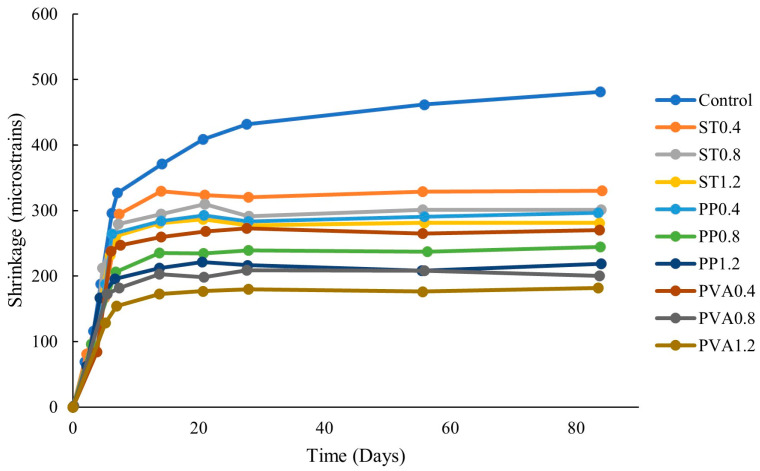
Drying shrinkage of slag-based concretes reinforced with steel fibers (Control = without fibers, ST0.4 = steel fiber content of 0.4%, PP0.4 = polypropylene fiber content of 0.4%, PVA0.4 = PVA fiber content of 0.4%) [33].

The characteristics of fibers significantly impact the drying shrinkage behavior of fiber-reinforced composites. The hydrophilic nature of PVA reduces moisture loss, which decreases drying shrinkage rates. Figure 14 shows the variation in drying shrinkage of slag-based concrete reinforced with various contents of PVA, steel, or polypropylene fibers [33]. It is noted that drying shrinkage increased significantly in the first 21 days, with limited change thereafter. All samples had drying shrinkage rates below 500 microstrains, which aligns with ASTM C157 [122] standards. Increasing the fiber volume fraction effectively decreased the drying shrinkage rates regardless of the type of fiber [33]. The addition of 1.2% PVA fiber content led to the highest enhancement in drying shrinkage rates compared to steel and polypropylene at the same content. This is due to their hydrophilic nature, which makes them interact with water more favorably than other fibers [33].

Reinforcing fibers are effective in enhancing the dimensional stability of mortars and concretes, but that efficacy depends on the type of fiber and binder. Steel fibers decreased drying shrinkage of alkali-activated slag-silica mortars by 24% compared to alkali-activated slag-silica mortars without fibers [15]. This reduction in drying shrinkage is consistent for both short (6 mm) and long (13 mm) steel fibers when used at the same fiber dosage [15]. Puertas et al. [55] reported that adding 0.22% glass fibers to sodium silicate-activated slag mortar resulted in approximately 20% reduction in drying shrinkage. This reduction was comparable to the shrinkage rate observed in Ordinary Portland cement mortar [55].

Increasing fiber content, up to an optimum value, increases the efficiency of fibers in enhancing concrete drying shrinkage rates. Increasing polypropylene fiber volume fraction from 0.05 to 0.2% decreased drying shrinkage of concrete consisting of OPC, 85% OPC + 15% fly ash and 80% OPC + 20% fly ash [34]. OPC-based concrete without fly ash or polypropylene fibers experienced the highest drying shrinkage. The effect of increasing fiber content and/or fly ash content is a consistent decrease in drying shrinkage. The composite containing 0.2% polypropylene and 20% fly ash experienced the most significant reduction of 20% compared to OPC concrete without fibers. This observation aligns with the study by Kronlof [123], who concluded that the addition of polypropylene fibers helps with control of bridge cracks at the micro-level [123].

#### 5.1.2. Effect of Binder Composition on Shrinkage of Concrete

When added to alkali-activated slag-based concrete, macro-polypropylene fibers reduced shrinkage [17]. This is due to the unique characteristics of macropolypropylene fibers, which prevent crack initiation. The combination of both polypropylene fiber and fly ash synergistically result in a significant reduction in drying shrinkage [34]. This is because adding fly ash slows down the polymerization process of the composite, which reduces drying shrinkage rates.

### 5.2. Water Absorption

Composites with coarser pore structure have typically higher water permeability than those with a finer pore structure. High water permeability of fiber-reinforced composites enhances transport of harmful materials through concrete to reinforcing steel bars.

#### 5.2.1. Effect of Fiber Type and Content on Water Absorption of Concrete

The water permeability of concrete is influenced by the pore diameter and pore connectivity. Ziada [54] reported that adding 0.2% to 1.2% basalt fibers to alkali-activated fly ash/slag mortar containing basalt powder increased its water absorption from 9.04% to 17.99%. This is due to the absorbent nature and large surface area of basalt fibers, which promote pore connectivity, especially at higher volume fractions. Similarly, Sadrmomtazi [124] reported that water absorption of mortar increased by 33% and 57% compared to mixes without fibers, when basalt fiber content was 1% and 1.5%, respectively. Other studies also reported that the water absorption of the cementitious composites increased with the dosage of basalt fiber [124].

The addition of natural fibers can affect the water absorption rates of concrete. Bheel et al. [125] found that the water absorption of concrete increases when a combination of nylon and jute fibers were combined to reinforce the matrix. This is be attributed to the tendency of nylon and jute fibers to absorb more water than concrete [125]. The entrapped air in concrete due to fiber addition may also contribute to increased water absorption [125]. However, it should be noted that the increase in water absorption may also be associated with reduced workability and increased voids in concrete as a result of fiber addition. The utilization of superplasticizer has been found to counteract the adverse effects of fibers on the water absorption capacity of concrete [102,116].

Other studies have shown that steel fibers at a content of up to 1% of the mixture volume can reduce water absorption in concrete. This is attributed to the ability of steel fibers to impede pore connectivity and reduce the overall porosity of the concrete [126]. Ramezanianpour [127] found that the addition of up to an optimum content of 0.7 kg/m^3^ polypropylene fibers to concrete decreases water absorption. While the rate of reduction in water absorption decreases as fiber content exceeds the optimum value of 0.7 kg/m^3^, higher dosage, up to 4 kg/m^3^, produced concrete with lower water absorption than unreinforced concrete. The reduction in water penetration with the addition of polypropylene fibers is attributed to its ability to effectively block connected pores in the concrete matrix, resulting in a decrease in capillary porosity [127].

**Figure 15:**
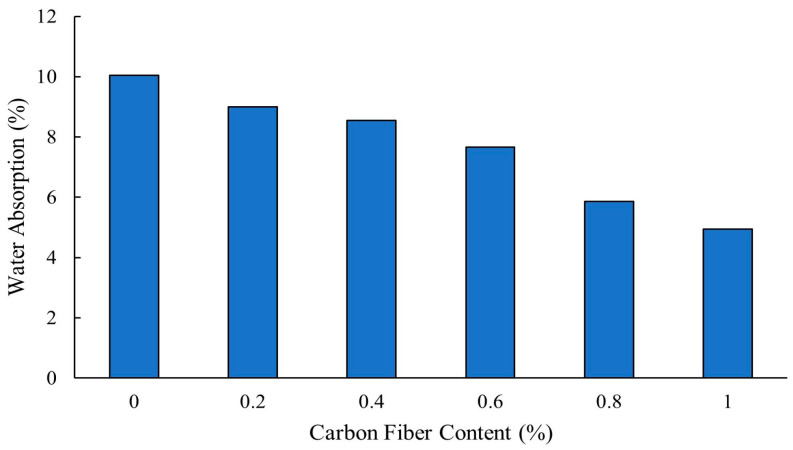
Water absorption of alkali-activated mortar reinforced with carbon fibers [32].

Incorporating carbon fibers into alkali-activated mortar decreases water absorption. Figure 15 shows the decrease in water absorption (%) as carbon fiber is increased to 1% [32].

#### 5.2.2. Effect of Curing Conditions on Water Absorption of Concrete

The curing method of fiber-reinforced concretes plays a critical role in water absorption characteristics [128]. Abdoellahnejad [47] reported that concrete reinforced with 0.5% PVA fiber and cured using a water bath exhibited the most significant reduction in water absorption.

### 5.3. Chloride Penetration in Concrete Reinforced with Fibers

Several damaging factors are present in harsh environments, including chloride ions, attacks from sodium sulfate and magnesium sulfate, salt crystallization, as well as abrasion and erosion [129]. Chloride ingress into concrete is one of the main causes of steel corrosion in reinforced concrete structures, which compromises durability and service life of reinforced concrete structures.

#### 5.3.1. Effect of Fiber Type and Content on Chloride Penetration of Concrete

Careful selection of appropriate reinforcing fiber type, geometry, and content can improve the chloride ion resistance of concrete. Adding basalt fibers, especially at higher dosages, to self-compacting concrete resulted in a decrease in chloride permeability [130]. The reduction in chloride permeability is attributed to the ability of basalt fibers to reduce the interconnected pores and overall porosity and permeability of concrete [131].

Steel reinforcements in concrete are typically protected from corrosion by a passive layer that forms on their surface in the highly alkaline pore solution of concrete. This passive layer can be destroyed due to the penetration of aggressive chloride ions. Wang et al. [132] proposed a model for evaluating chloride diffusion in steel fiber-reinforced concrete subjected to flexure. Under flexural loads, the chloride diffusion coefficient was found to be 40% lower than concrete without fibers [132].

When reinforcing fibers cause a reduction in chloride diffusion coefficient, it is associated the formation of a three-dimensional support system by the distributed fibers within the concrete matrix, which limits the generation and propagation of microcracks while also compressing the capillary pores in the mortar. Liu et al. [83] concluded that the inclusion of polypropylene fibers in high-performance geopolymer concrete positively influences its chloride resistance. The addition of polypropylene fibers at contents of 0.45 kg/m^3^, 0.9 kg/m^3^, and 1.35 kg/m^3^ decreased the chloride content and depth gradually, with 1.35 kg/m^3^ fiber content yielding the most significant effect. Similarly, Sukontasukkul [17] observed a reduction in chloride penetration depth and diffusivity with the inclusion of steel, polypropylene, and PVA fibers in contents ranging from 0.5% to 1.5%.

Aisheh [12] reported that chloride permeability of alkali-activated slag-microsilica concretes with a combination of 2% steel and 0.25% polypropylene fibers was assessed to be very low based on testing according to ASTM C1202 [133]. The alkali-activated concrete with steel and polypropylene fibers exhibited a 47% reduction in passing charge compared to the composite without fibers. The is because polypropylene fibers have lower conductivity compared to steel fibers, which contributes to a reduction in the passing flow of chloride ions through the concrete [12].

Ali [116] reported that incorporating glass fibers increased chloride permeability of concrete by 11%. Glass fibers increased the pore connectivity of concrete and provided more pathways for chloride ions to penetrate. However, the inclusion of fly ash is known to enhance resistance of concrete to chloride migration [134] and therefore mitigate the adverse effect of reinforcing glass fiber [116]. Chalah and Talah [135] reported that in the long term, reinforcing high performance concrete with polypropylene fibers does not affect the chloride resistance of concrete. The electrical charge passed through both high-performance concrete, with and without fibers, and becomes nearly comparable after a 90-day exposure period [135]. However, Hu [136] found that the addition of polypropylene fibers had a more pronounced impact on reducing chloride resistance compared to basalt fibers. Combining both types of fibers did not lead to an improvement; instead, the reduction in chloride resistance became more significant at higher volumes of hybrid fibers [136].

Gulzar [102] reported that the chloride ion penetration depth of plain concrete increased by 27% with the addition of 0.5% jute fiber when samples were immersed in a 10% NaCl aqueous solution. Jute fibers increased the porosity of the composite network. However, the addition of superplasticizer reduces the chloride ion penetration depth of 0.5% jute fibers composite by around 17% compared to the control mix [102]. This reduction is attributed to the ability of the superplasticizer to improve compaction and reduce voids created by tangling and accumulation of fiber filaments [102].

#### 5.3.2. Effect of Binder Composition

Partial replacement of OPC with up to 40% fly ash enhanced resistance of self-consolidating concrete to chloride migration. In addition, resistance of self-consolidating concrete to chloride penetration in which 40% of OPC was replaced with fly ash and was enhanced even more when 1% to 2% basalt fibers was added to the mix [137].

Chen [138] proposed a numerical model to predict the service life of geopolymer mortars reinforced with BFRP bars in marine conditions.

Table 5 summarizes studies that evaluated the long-term durability of fiber-reinforced concretes in corrosive, acidic, and dry environments.

## 6. Conclusions

Various types of reinforcing fibers, including but not limited to steel, basalt, PVA, polypropylene, and natural fibers, enhance durability and strength of concrete with alkali-activated binders and other types of binders. This article reviewed the behavior of fiber-reinforced composites. The main findings are:

- Studies evaluated concrete/mortar reinforced with fiber volume fractions in the range of 0.1% to 5%. Lower fiber content, in the range of 0.1% to 0.3%, controls stresses arising from high temperatures and shrinkage. Fiber content higher than 0.3% enhances the load-carrying capacity and post-cracking behavior of concretes.
- The addition of reinforcing fibers increases the interactions between the fibers and the concrete matrix, hindering the flow of concrete. Longer fibers may cause clustering and non-uniform distribution in the matrix, leading to higher flow reductions compared to shorter fibers. At the same fiber dosage, hooked-end steel fibers enhance the flow characteristics compared to straight and corrugated fibers.
- High content of rigid steel fibers may produce highly stiff concrete, especially when added at a volume fraction exceeding 1.5%. The hydrophobicity of polypropylene fibers, as indicated by its low water absorption, may also lead to less workable concrete mixes. A fiber dosage of 0.5% steel, 0.3% basalt, or 0.1% polypropylene produced concrete within the required slump range.
- The concentration and type of the alkaline activator influence the workability of fiber-reinforced concretes, but their effect is less significant than fiber type, dosage, and geometry. A high sodium silicate-to-sodium hydroxide ratio increases the viscosity of the mixture, resulting in slower concrete flow. On the other hand, a high water-to-binder ratio enhances the flowability of fiber-reinforced concrete/mortar.
- The stiffness of reinforcing fibers plays an important role in compressive behavior of fiber-reinforced composites. Fibers with high elastic moduli distribute internal compressive stresses uniformly and decrease stress concentration and local fractures, while fibers with low elastic moduli increase compressibility and local fractures.
- The effectiveness of reinforcing fibers is strongly dependent on the interaction of fibers with the matrix in the interfacial transition zone. Strong interfacial bond leads to higher load-bearing capacity. The strength of the bond between reinforcing fibers and geopolymer matrix is typically higher than the bond between fibers and cement composites, resulting in a significant improvement in flexural strength of geopolymer concrete.
- Increasing fiber contents, up to specific optimum values, enhances the tensile and flexural strengths of alkali-activated and conventional concrete by improving the homogeneity of the matrix. An excessive amount of fibers can make the concrete matrix porous and heterogeneous, which negatively impacts mechanical properties.
- Alkali-activated slag-based concrete reinforced with steel fibers exhibited improved performance compared to fly ash-based and conventional concretes. Workability decreases with the addition of steel fibers but remains within acceptable practicable ranges when the fiber volume fractions is around 0.5%.
- Long fibers have a greater bonding surface area with the concrete matrix than shorter length fibers. Therefore, up to an optimum content, long fibers are more effective in transferring tensile stresses in the tension zone. Beyond the optimum content, long fibers decrease the homogeneity of concrete, introduce voids, and decrease mechanical properties.
- The addition of an appropriate dosage of hybrid reinforcing fibers, such as the combination of steel and polypropylene, was shown to improve splitting tensile strength and flexural strength. The increase in tensile strength contributes to controlling the inception and propagation of cracks.
- Fiber-reinforced concrete exhibited enhanced post-cracking behavior and has high ductility and energy absorption characteristics compared to unreinforced concrete. The energy absorption during crack propagation is influenced by the interactions between fibers and the matrix, which include bridging, de-bonding, pullout, and rupture.
- Water absorption and chloride penetration of fiber-reinforced concretes are highly dependent on the type of fibers used. The absorbent nature and moisture sensitivity of natural fibers such as jute, sisal, and cotton generally increase the water absorption and chloride penetration of concrete.
- The volumetric shrinkage and stability of alkali-activated and conventional concretes are controlled with the addition of fibers. The effectiveness of fibers in reducing concrete shrinkage depends on fiber type, content, and modulus of elasticity. High-stiffness fibers, such as polyvinyl alcohol (PVA), steel, and polypropylene, prevent crack localization induced by drying shrinkage.

## Figures and Tables

**Table 2:** Studies on the workability/flowability of fiber-reinforced alkali-activated composites.

Binder Type	Fiber Characteristics (Dimensions, Volume Fractions)	Selected Research Findings
GGBS + 30% Microsilica (Na_2_SiO_3_ + NaOH, w/b = 0.33) [12]	- Steel: 15 mm × 0.12 mm, 1% to 2.25%. - Polypropylene fiber: 8 mm × 0.033 mm, 0% to 0.25%.	Addition of 2.25% steel fiber content into concrete reduced the slump from 190 mm to 130 mm. The slump further reduced to 120 mm when polypropylene fibers were added alongside steel fibers.
Fly ash + 30% + GGBS (Na_2_SiO_3_ + NaOH, w/b = 0.4) [13]	- Steel: 6 mm × 0.15 mm, 0.5% to 2.5%. - Polypropylene: 9 mm × 0.0074 mm, 0.5% to 2.5%.	Addition of 2.5% steel fiber content into alkali-activated concrete decreased the slump from 115 mm to 50 mm. When 2.5% polypropylene fibers were added, slump decreased from 115 mm to 55 mm.
100% slag (Na_2_SiO_3_ + NaOH) [14]	- Steel: 6 mm × 0.75 mm, 0% to 2.5%. - Steel: 12 mm × 0.75 mm, 0% to 2.5%.	Addition of 2.5% short steel fiber content into slag-based concrete reduced the flow diameter from 160 mm to 120 mm. Addition of longer steel fibers decreased the diameter from 160 mm to 110 mm.
GGBS + 20% silica fume (Na_2_SiO_3_ + NaOH, w/b = 0.17) [15]	- Brass-coated steel: 6 mm and 13 mm × 0.16 mm, 0.5% to 2%.	Addition of 2% short steel fiber into alkali-activated concrete reduced the flow diameter from 157 mm to 140 mm. Addition of longer steel fibers decreased the diameter from 157 mm to 119 mm.
OPC + 20% fly ash [34]	- Polypropylene: 0.05% to 0.2%.	Addition of 0.2% polypropylene fiber into OPC concrete reduced the slump from 170 mm to 140 mm. Partial replacement of OPC with 20% fly ash increased slump to 190 mm.
100% fly ash (Na_2_SiO_3_ + NaOH) [68]	- High-strength steel: 13 mm × 0.2 mm - Polyvinyl alcohol (PVA): 8 mm × 0.04 mm. - Polypropylene: 12 mm × 0.05 mm. - Polyester: 6 mm × 0.03 mm. - Carbon: 10 mm × 0.015 mm.	High-strength steel fibers (0.5% and 1%) had a minimal impact on flow. Polyvinyl alcohol (0.5% and 1%), polypropylene (0.5% and 1%), carbon, and polyester fibers (0.5% and 1%) caused a reduction in flow, with 0.5% polyester microfibers causing the most reduction.
OPC + microsilica (w/b = 0.41) [83]	- Polypropylene: 12 mm × 35 µm. Fiber content: 0.45 kg/m^3^, 0.9 kg/m^3^, 1.35 kg/m^3^.	Slump decreased with the increase in polypropylene fiber content. At 1.35 kg/m^3^ polypropylene fiber content, the slump reduced from 180 mm to 155 mm.
Fly ash + 20% GGBS (Na_2_SiO_3_ + NaOH, w/b = 0.4, 0.45) [17]	- Hooked-end steel: 35 mm × 0.55 mm, 0%, to 1.5%.	Addition of 1.5% hooked-end steel fiber content into concrete decreased the flow diameter from 797 mm to 735 mm. Concrete mixes with a higher w/b ratio exhibited better flowability, while higher NaOH concentrations reduced flowability.
Fly ash + 35% GGBS (Na_2_SiO_3_ + NaOH, w/b = 0.55) [18]	- Basalt: 12 mm × 0.020 mm, 0.1% to 0.35%. - Steel: 12 mm × 0.020 mm, 0.4% to 0.6%. - Polypropylene: 12 mm × 0.020 mm, 0.05% to 0.25%.	As the NaOH molarity increases, the workability of the concrete consistently decreases with all types of fibers. For 8 M and 10 M NaOH concentrations, the optimal fiber dosages for the required slump range were 0.3% for basalt fiber, 0.55% for crimped steel fiber, and 0.1% for polypropylene fiber.
OPC + microsilica [32]	- Carbon: length = 10 mm, 0.2% to 1%.	The initial slump without fibers was 711 mm. As the carbon fiber volume fraction increased to 1%, the slump values decreased by 11.92%, reaching 630 mm.
OPC + fly ash + GGBS + microsilica [19]	- Hooked-end steel: 35 mm × 0.55 mm, 0.5% to 0.1%. - Micro-steel: 10 mm × 0.12 mm, 0.5%, to 0.1%.	Addition of 0.5% hooked steel fibers and 0.25% micro-steel fibers decreased the slump values for both the self-compacting concrete with a strength grade of 80 MPa and that with a strength grade of 60 MPa.
100% fly ash (Na_2_SiO_3_ + NaOH) [35]	- Polypropylene: 12 mm × 34 µm, 0% to 0.2%.	Addition of polypropylene fiber into fly ash-based concrete reduced the flow. The highest reduction occurred with 0.2% polypropylene fiber content.
Slag + fly ash + silica fume (Na_2_SiO_3_ + NaOH, w/b = 0.32) [20]	- Steel (corrugated, hooked-end, and straight): 13 mm × 0.12 mm, 0%, to 3%.	Without fiber, the flow diameter of concrete reached 260 mm. Adding corrugated steel fibers at volume fractions up to 3% decreased the flowability by a maximum of 6.9%. In comparison to straight and corrugated steel fibers, hooked-end steel fibers exhibited higher flowability at fiber content of 2% and 3%.
OPC + natural zeolite [61]	- Polyolefin: 0.25% to 1.25%.	As the content of polyolefin fiber is increased from 0% to 1.25%, the slump flow diameter decreased and the T50 time increased.
OPC + nanosilica (w/b = 0.35) [36]	- Glass: 1 mm × 13 µm, 0% to 1.5%.	Addition of 1.5% glass fiber content to concrete decreased the slump flow. A combination of nanosilica and glass fibers caused further decrease in flow.
OPC + 10% fly ash and OPC + 10% silica fume [84]	- Basalt: 12 mm × 13 µm, 0.1% to 0.4%.	Replacement of fly ash into basalt fiber-reinforced OPC concrete increased the slump from 90 mm to 120 mm, while the replacement of silica fume reduced the slump.
50% fly ash, 50% slag (Na_2_SiO_3_ + NaOH) [16]	- Straight steel: 6 mm × 0.2 mm, 1% to 3%. Deformed steel: 18 mm × 0.55 mm, 1% to 3%. Hybrid steel content: 2%.	The incorporation of combined microsteel and deformed steel fibers at a volume fraction of 2% led to the most significant reduction in slump compared to their individual use. The slump flow decreased from 118 mm to 75 mm.

**Table 3:** Studies on the compressive strength of fiber-reinforced alkali-activated composites.

Binder Type	Fiber Characteristics and Content	Curing Age	Selected Research Findings
GGBS + fly ash (Na_2_SiO_3_ + NaOH, SiO_2_/Na_2_O = 1.63, w/b = 0.55) [22]	- Corrugated steel: 36 mm × 1.08 mm. Vol. fractions: 0%, 0.5%, and 1.4%.	Ambient curing for 28 days.	Addition of 1.4% corrugated steel fibers into alkali-activated GGBS/fly ash concrete increased the elastic modulus to 30.6 GPa and compressive strength to 70.8 MPa.
Fly ash + 25% rice husk ash +10% GGBS (Na_2_SiO_3_ + NaOH, Na_2_SiO_3_/NaOH = 2.5) [70]	- Basalt: 6 mm × 13–20 µm. Vol. fractions: 1%, 2%, 3%, and 4%.	Cured for 28 days. Values are taken at 3, 7, 28 days.	Addition of 2% basalt fiber into concrete alkali-activated concrete with fly ash, RHA, and GGBS increased the compressive strength to 28 MPa.
70% GGBS + 30% microsilica (Na_2_SiO_3_ + NaOH, Na_2_SiO_3_/NaOH = 3.5, w/b = 0.33) [12]	- Steel: 15 mm × 0.12 mm. Vol. fractions: 1% to 2.25%. - Polypropylene: 8 mm × 0.033 mm. Vol. fractions: 0% to 0.25%.	Ambient curing for 28 days.	The highest increase in compressive strength occurred with the addition of 2.25% steel fiber content. In addition, 0.25% polypropylene fibers along with 2% steel fibers increased the compressive strength to 154 MPa.
90% fly ash + 10% GGBS (Na_2_SiO_3_ + NaOH) [66]	- Basalt: aspect ratio of 20: 0% to 1%. - Polypropylene: aspect ratio of 12: 0% to 2%. - Hooked-end steel fiber: 0% to 2%.	Ambient curing for 24 h then oven cured at 80 °C for 24 h.	Addition of 0.2% of basalt fibers increased the compressive strength to 67 MPa, 0.8% of polypropylene fibers to 67 MPa, and 1% of steel hooked-end fibers to 73 MPa. Meanwhile, 1% steel fibers led to the highest reduction.
Fly ash + GGBS (NaOH + Na_2_SiO_3_, Na_2_SiO_3_/NaOH = 2.5, w/b = 0.4) [13]	- Steel: 6 mm × 0.15 mm: 0.5% to 2.5%. - Polypropylene: 9 mm × 0.0074 mm: 0.5% to 2.5%.	Ambient curing for 28 days.	Addition of up to 2.5% steel fibers in alkali-activated concrete led to 13.6% increase in compressive strength to reach 65 MPa. When 2% polypropylene fibers were included, the compressive strength increased to 55 MPa.
100% slag (12 M NaOH + Na_2_SiO_3_, Na_2_SiO_3_/NaOH = 2.5 and 3) [14]	- Steel: 6 mm × 0.75 and 12 mm × 0.75 mm. Vol. fraction: 0% to 2.5%.	Ambient and heat curing for 28 days.	Addition of 2.5% short steel fibers led to the highest compressive strength of 80 MPa, while longer fibers experienced a lower compressive strength of 70 MPa.
100% fly ash (14 M NaOH + Na_2_SiO_3_, Na_2_SiO_3_/NaOH = 2.5) [25]	- Hooked-end steel: aspect ratio of 65. Vol. fractions: 0% to 0.5%.	Heat curing for 24 h at 65 °C followed by air curing for 28 days.	Addition of 0.5% hooked-end steel fibers to fly ash-based concrete led to a 7% increase in compressive strength.
80% slag + silica fume (NaOH + Na_2_SiO_3_, SiO_2_/Na_2_O = 1.2, w/b = 0.17) [15]	- Brass-coated steel: 6 mm and 13 mm × 0.16 mm diameter: 0% to 2%.	Heat and steam curing for 28 days.	Addition of 2% short steel fibers (6 mm in length) increased the compressive strength to 195 MPa, while the addition of 2% long steel fibers (13 mm in length) increased the compressive strength to 229 MPa.
100% slag (Na_2_SiO_3_, SiO_2_/Na_2_O = 1.18, w/b = 0.56) [55]	- AR-glass: 12 mm × 14–20 µm diameter: 0.11%, 0.22%, and 1.1%.	Air curing for 28 days.	Compressive strength of both Ordinary Portland cement and sodium silicate-activated slag mortars were not affected by the addition of 0.11% to 1.1% glass fiber contents.
100% Fly ash (12 M and 16 M NaOH) [56]	- Glass: aspect ratio of 600. Vol. fractions: 0.1% to 0.5%.	Normal and thermal curing.	Addition of 0.3% glass fiber content in thermally cured fly ash-based concrete activated with 16 M of sodium hydroxide increased the compressive strength to 24.8 MPa.
100% fly ash (NaOH + Na_2_SiO_3_, SiO_2_/Na_2_O = 3.2, w/b = 0.4) [37]	- Basalt: lengths of 3 mm, 6 mm, 12 mm, and 18 mm. Vol. fractions: 0% to 0.1%.	Heat curing for 16 h.	Addition of 0.1% volume fraction of 6 mm basalt fibers to fly ash-based concrete led to the highest compressive strength of 49.85 MPa.
70% fly ash + 30% slag (NaOH + Na_2_SiO_3_, SiO_2_/Na_2_O = 1.5, w/b = 0.4) [26]	- Polypropylene: 12 mm × 18–30 µm: 0% to 0.5% - Basalt: 12 mm × 7–30 µm: 0% to 0.5% - Steel: 13 mm × 0.2 mm: 0% to 0.5%	Curing for 24 h and demold until 28 days.	Addition of 0.2% polypropylene, 0.4% basalt, and 0.5% steel fibers independently led to optimum increases in compressive strength by 9.9%, 18.5%, and 22.9%, respectively.
60% fly ash + 40% slag (NaOH + Na_2_SiO_3_, NaOH:Na_2_SiO_3_ = 1:2.5) [41]	- Polypropylene: 12 mm × 40 µm: 0% to 5%.	Ambient curing for 28 days.	Addition of 2% polypropylene fiber content into alkali-activated concrete containing 60% fly ash and 40% slag increased the compressive strength to 76 MPa.
60% fly ash + 40% slag (NaOH + Na_2_SiO_3_, SiO_2_/Na_2_O = 1.2, w/b = 0.28) [39]	- PVA: 8 mm × 40 µm: 0.5% to 2%. - PVA: 12 mm × 100 µm: 0.5% to 2%.	Standard curing for 28 days.	Addition of 2% short PVA fiber content (8 mm in length) increased the compressive strength from 49.18 MPa to 84.95 MPa. Meanwhile, 2% long PVA fiber content increased the compressive strength to 68.36 MPa.
OPC + 15% fly ash OPC + 20% fly ash [34]	- Polypropylene: Vol. fractions: 0% to 0.2%.	Ambient curing for 28 days.	Addition of polypropylene fiber across volume fractions ranging from 0% to 0.2% does not yield any substantial impact on both compressive strength and elastic modulus.
100% slag (12 M NaOH + Na_2_SiO_3_, Na_2_SiO_3_/NaOH = 2.5) [90]	- Hooked-end steel: 50 mm × 0.8 mm. - Polypropylene: 48 mm × 0.85 mm	Ambient curing for 28 days.	Compressive strength increased from 40.5 MPa to 44.5 MPa with the addition of 2% polypropylene fibers. The addition of 2% steel fibers increased the compressive strength by 9.11% to reach 44 MPa.
100% slag (Na_2_SiO_3_ with Na_2_O% = 5%, w/b = 0.45) [27]	- Steel weights: 40 kg/m^3^, 120 kg/m^3^. Vol. fractions: 0% to 1.2%.	Ambient cured for 28 days.	Addition of 1.2% steel fibers to slag-based concrete and OPC caused a decrease in compressive strength, with alkali-activated slag concrete showing more significant declines.
100% slag (Ca(OH)_2_ + Na_2_SO_4_, w/b = 0.34) [93]	- Polyethylene: 18 mm × 12 µm. Vol. fractions: 0% to 1.75%.	Ambient cured for 28 days.	Addition of 1.75% of polyethylene fiber in slag-based concrete exhibited a compressive strength of 54.8 MPa.
100% slag (NaOH + Na_2_SiO_3_, w/b = 0.34, 0.38, 0.44) [98]	- PVA: 12 mm × 39 µm: 0% to 2%.	Ambient curing for 28 days.	Addition of 2% PVA fiber to slag-based concrete with the lowest w/b ratio of 0.34 achieved the highest compressive strength of 30.6 MPa at 28 days.
100% fly ash (NaOH + Na_2_SiO_3_, w/b = 0.4) [97]	- PVA: 18 mm × 12 µm: 0% to 2%.	Heat curing for 28 days.	Addition of 2% of PVA fibers in fly ash-based concrete for a longer period (8 h) compared to a shorter period (4 h) resulted in the highest compressive strength of 27.6 MPa.
100% slag (NaOH + Na_2_SiO_3_, w/b = 0.4) [100]	- PVA: 18 mm × 12 µm: 0% to 0.02%.	Air cured for 28 days.	Slag-based concrete reinforced with 0.02% of PVA fiber exhibited the highest compressive strength of 63.7 MPa.
100% fly ash (12 M NaOH + Na_2_SiO_3_) [33]	- Steel fiber: 6 mm × 0.17 mm: 0% to 1.2%. - Polypropylene: 12 mm × 0.0075 mm. Vol. fractions: 0% to 1.2% - PVA: 8 mm × 0.04 mm.	Heat cured at 80 °C for 24 h. Values are taken at 28 days.	The enhancement in compressive strength was relatively minor, as the 28-day compressive strength of composites containing steel and polyvinyl alcohol fibers increased by 3.37% and 4.26%, respectively.
100% fly ash (NaOH + Na_2_SiO_3_, w/b = 0.32) [40]	PVA	Steam curing at 80 °C and standard curing at 20 °C.	The compressive strength of geopolymer mortar exhibited an increase in compressive strength with increasing PVA fiber content up to an optimum value of 0.8%.
Cement + fly ash + slag (NaOH + Na_2_SiO_3_, w/b = 0.41) [83]	- Polypropylene: 12 mm × 35 µm.	Ambient curing for 24 h.	Polypropylene fibers had minimal impact on compressive strength after 7 days of curing, displaying a similar strength to unreinforced composites. However, after 28 days, the compressive strength became higher.
Fly ash + 20% slag (8 M, 12 M NaOH + Na_2_SiO_3_, NaOH/Na_2_SiO_3_ = 1, w/b = 0.4, 0.45) [17]	- Hooked-end steel: 35 mm × 0.55 mm. Vol. fractions: 0%, 0.5%, 1%, 1.5%.	Curing at room temperature for 28 days.	Addition of 1.5% polypropylene fibers in 12 M sodium hydroxide-activated fly ash/slag concrete with a water-to-binder ratio of 0.4 resulted in a maximum compressive strength of 64 MPa.
Metakaolin + fly ash (K_2_SiO_3_, SiO_2_/K_2_O molar ratios of 1.0) [101]	- Cotton: 6 mm × 7 µm. Vol. fractions: 2% w/b= 0.4, 0.5.	Room temperature curing.	Incorporation of 2% cotton fiber into a mix consisting of 50% metakaolin and 50% fly ash increased the compressive strength. This increase occurred after exposure to 100 °C. Strength decreased when exposed to temperature in the range of 100–800 °C.
GGBS + fly ash (8 M, 10 M NaOH + Na_2_SiO_3_, Na_2_SiO_3_/NaOH = 2.5) [18]	- Basalt: 12 mm × 13 µm. Vol. fractions: 0.1% to 0.35%. - Crimped steel: 25 mm × 0.5 mm. Vol. fractions: 0.4% to 0.6%. - Polypropylene: 12 mm × 0.038 mm. Vol. fractions: 0.05% to 0.25%.	Ambient curing.	Alkali-activated concrete with basalt fibers had the highest compressive strength at both 8 M and 10 M NaOH concentrations, followed by steel fibers, while polypropylene fibers exhibited comparatively lower compressive strength.
Cement + microsilica [32]	- Carbon: 10 mm long: content: 0.2%, 0.4%, 0.6%, 0.8%, 1%.	Values were recorded for 28 days of curing.	Adding carbon fiber up to an optimum content of 0.6% increased the compressive strength with a maximum of 45.342 MP. Increasing the content of carbon fiber beyond 0.6% decreased strength.
Cement [102]	- Jute: 10 mm in length: 0.2% to 1%.	Cured in tap water for 28 days. Then conditioned indoors.	The addition of 0.5% volume of jute fiber and superplasticizer increased compressive strength by 13.1%.
Metakaolin + fly ash (NaOH + Na_2_SiO_3_, Na_2_SiO_3_/NaOH = 6.27, w/b = 0.65) [40]	- PVA: 12 mm × 40 μm. Vol. fractions: 0% to 1.2%.	Curing at 25 °C and 200 °C.	At temperatures between 25 °C and 200 °C, the addition of 0.8% PVA fiber increased the compressive strength by 35.6% and 50.5%, respectively. However, when temperatures exceeded 200 °C, the compressive strength decreased.
Cement + GGBFS + fly ash + microsilica [19]	- Hooked-end steel: 35 mm × 0.55 mm. - Microsteel: 10 mm × 0.12 mm: 0.1% to 0.5%.	Moist curing for 7, 14, and 28 days.	The combination of hooked steel and microsteel fibers, with volume fractions of 0.4% and 0.1%, led to a 7.89% increase in compressive strength for 60 MPa concrete and a 2.90% increase for 80 MPa concrete.
100% fly ash (NaOH + Na_2_SiO_3_) [35]	- Propylene: 12 mm × 34 µm: 0% to 0.2%.	Curing at 80 °C for 24 h.	The addition of polypropylene microfibers had a limited impact on the compressive strength of fly ash-based concrete but decreased the elastic modulus.
Slag + fly ash + silica fume (NaOH + Na_2_SiO_3_) [20]	- Corrugated steel. - Hooked-end steel: 13 mm × 0.12 mm. - Straight steel: 6, 8, and 13 mm × 0.12 mm.	Steam curing at 80 °C and standard curing at 20 °C.	The highest elastic modulus of 31.5 GPa, which was 22.1% higher than the modulus of the composite without steel fibers, was achieved with the use of 3% steel fibers.
Fly ash + slag + basalt powder (12 M NaOH + Na_2_SiO_3_) [54]	- Basalt: 12 mm × 0.02 mm. Vol. fractions: 0.4%, 0.8%, and 1.2%.	Curing at 60 °C for 24 h then at 20 °C for 28 days.	The addition of 1.2% basalt fibers led to a 11.94% increase in compressive strength. The inclusion of basalt fibers led to improvements in compressive strengths ranging from 9.01% to 15.79% across all basalt fiber contents.
OPC + fly ash OPC + fly ash + silica fume [103]	- Basalt: 12 mm in length: 0% to 0.6%.	Standard curing at 20 ± 1 °C until testing at 28 and 56 days.	The compressive strength exhibits an increase with fiber content, followed by a subsequent decrease as the volume content of basalt fiber increases beyond a threshold 0.15%.
OPC + silica fume + fly ash + slag [104]	- Basalt: 18 mm × 15 µm: 0% to 0.15%. - Propylene: 19 mm × 30 µm: 0% to 0.15%.	Cured at 20 °C and 98% relative humidity.	Adding 0.15% basalt fibers and 0.15% polypropylene fibers enhanced the compressive strength for both C30 and C40 strength grades. Amounts of 0.15% basalt fibers and 0.1 polypropylene fibers performed best for C50 strength grade. Exceeding 0.15% fiber content results in a decrease in compressive strength.

**Table 4:** Studies on the splitting tensile and flexural strength of fiber-reinforced alkali-activated composites.

Binder Type	Fiber Characteristics and Content	Curing Age	Result
GGBS + fly ash (Na_2_SiO_3_ + NaOH, SiO_2_/Na_2_O = 1.63, w/b = 0.55) [22]	- Steel: 36 mm × 1.08 mm: 0% to 1.4%.	Ambient curing for 28 days.	Addition of 1.4% corrugated steel fiber content increased the toughness factor from 1.32 to 1.82, indicating 1.5 times increase in ductility.
Fly ash + 25% rice husk ash +10% GGBS (Na_2_SiO_3_ + NaOH, Na_2_SiO_3_/NaOH = 2.5) [70]	- Basalt: 6 mm × 13–20 µm. Vol. fractions: 1%, 2%, 3%, and 4%.	Cured for 28 days. Values are taken at 3, 7, 28 days.	Addition of 2% basalt fiber into alkali-activated concrete increased the tensile strength by 23.98% and flexural strength by 43%. Flexural strength increased from 6 MPa to 7 MPa.
70% GGBS + 30% microsilica (Na_2_SiO_3_ + NaOH, Na_2_SiO_3_/NaOH = 3.5, w/b = 0.33) [12]	- Steel: 15 mm × 0.12 mm: 1% to 2.25%. - Polypropylene: 8 mm × 0.033 mm. Vol. fractions: 0% to 0.25%.	Ambient curing for 28 days.	Concrete containing 2.25% steel fibers demonstrated the highest tensile (7.7 MPa) and flexural strength (13.7 MPa) at 28 days. The composite with 2% steel fibers and 0.25% polypropylene fibers exhibited a splitting tensile strength of 8.4 MPa and flexural strength of 13.6 MPa.
90% fly ash + 10% GGBS (Na_2_SiO_3_ + NaOH) [66]	- Basalt: aspect ratio (AR) of 20: 0% to1%. - Polypropylene: AR = 12: 0% to 2%. - Hooked-end steel: AR = 30: 0% to 2%.	Ambient and oven curing.	Addition of 0.2% of basalt fibers increased tensile strength to 2.9 MPa, 0.8% of polypropylene fibers to 2.7 MPa, and 1% of steel hooked-end fibers to 4.5 MPa. Basalt fiber displays no post-peak response, whereas polypropylene fiber composite showed enhanced behavior when content exceeds 0.6%. Meanwhile, steel fiber exhibits a gradual post-peak response.
Fly ash + GGBS (NaOH + Na_2_SiO_3_, Na_2_SiO_3_/NaOH = 2.5, w/b = 0.4) [13]	- Steel: 6 mm × 0.15 mm: 0.5% to 2.5%. - Polypropylene: 9 mm × 0.0074 mm: 0.5%, 1.0%, 1.5%, 2.0%, and 2.5%.	Ambient curing for 28 days. Values were reported for 7 and 28 days.	Incorporating up to 2.5% steel fibers improved the splitting tensile strength by 52% to reach 9 MPa and flexural strength by 57.79% to reach 13 MPa. Concrete with 2% polypropylene fiber had similar tensile strength of 7.5 MPa and flexural strength of 10 MPa. The flexural toughness increased as the fiber content was increased.
100% slag (12 M NaOH + Na_2_SiO_3_, Na_2_SiO_3_/NaOH = 2.5 and 3) [14]	- Steel: 6 mm × 0.75 mm. Vol. fractions: 0% to 2.5%. - Steel: 12 mm × 0.75 mm. Vol. fractions: 0% to 2.5%.	Ambient and heat curing for 28 days.	Addition of 2.5% short steel fibers with Na_2_SiO_3_/NaOH = 2.5 led to increase in flexural strength reaching 17 MPa, while longer fibers experienced a higher flexural strength of 20 MPa. As subjected to elevated temperatures, the flexural strength of concrete increased with both short and long fibers.
100% fly ash (14 M NaOH + Na_2_SiO_3_, Na_2_SiO_3_/NaOH = 2.5) [25]	- Hooked-end steel: aspect ratio of 65. Vol. fractions: 0% to 0.5%.	Heat cured for 24 h at 65 °C then air cured for 28 days.	Addition of 0.5% hooked-end steel fibers to fly ash-based concrete led to an 8% increase in flexural strength and 57% increase in splitting tensile strength. The toughness increased when 0.5% hooked-end steel fibers was added compared to the mix without fibers.
100% fly ash (8 M NaOH + Na_2_SiO_3_ NaOH:Na_2_SiO_3_ = 0.4:1) [113]	- Steel: 10 mm × 0.12 mm: 0%, to 2%. - Polyvinyl alcohol: 8 mm × 0.04 mm. Vol. fractions: 0%, 1%, 2%.	Steam cured at 60 °C after casting for 24 h then stored in lab.	Addition of up to 2% of steel fibers to fly ash-based concrete enhanced the modulus of rupture (MOR) compared to PVA fibers but resulted in decreased deflection capacity due to its high modulus. The addition of up to 2% PVA fibers in fly ash-based concrete enhanced deflection capacity compared to steel fibers.
100% slag (Na_2_SiO_3_, SiO_2_/Na_2_O = 1.18, w/b = 0.56) [55]	- AR-glass: 12 mm × 14–20 µm. Vol. fractions: 0.11%, 0.22%, 1.1%.	Air curing for 28 days.	The flexural strength of both Ordinary Portland cement and sodium silicate-activated slag mortars were not affected by the addition of glass fibers of volume fractions of 0.11%, 0.22%, and 1.1%.
100% fly ash (12 M and 16 M NaOH) [56]	- Glass: aspect ratio of 600. Vol. fractions: 0.1%,0.2%,0.3%,0.4%, and 0.5%.	Normal and thermal curing for 7 and 28 days.	The addition of 0.3% glass fiber content in thermally cured fly ash-based concrete activated with 16 M of sodium hydroxide resulted in an increased splitting tensile strength and flexural strength to 1.6 MPa and 6 MPa, respectively.
OPC+ metakaolin + fly ash (K_2_SiO_3_ + KOH w/b = 0.4 and 0.5) [101]	- Short carbon: 6 mm × 7 µm. Vol. fractions: 0%, 0.2%.	Ambient curing for 28 days. Thermal curing for 28 days.	Addition of 0.2% short carbon increased the flexural strength of concretes cured at room and elevated temperatures. Initial strength increases at 100 °C; however, subsequent exposure within the 100 °C to 800 °C range led to some strength degradation, particularly noticeable in alkali-activated concrete in comparison with OPC.
Ladle slag + metakaolin (8 M NaOH + Na_2_SiO_3_ SiO_2_/Na_2_O = 1.99) [109]	- Carbon HT: 7 ± 1 mm × 10 µm. - E-glass fiber: 7 ± 1 mm × 10 µm.: 0%, 1%. - PVA fiber: 7 ± 1 mm × 10 µm: 0% to 1%. - PVC fiber: 7 ± 1 mm × 10 µm.	Ambient curing. Values are taken at 28 days.	Addition of 1% carbon fiber, glass fiber, PVA, or PVC into alkali-activated concrete led to increase in flexural strength for all the fiber-reinforced samples. The strength improvement ranged from 30% to 70% in comparison to the alkali-activated concrete without fibers. Carbon fibers had the highest increase in flexural strength and post-cracking behavior, resulting in high fracture toughness and increased ductility.
100% fly ash (NaOH + Na_2_SiO_3_, SiO_2_/Na_2_O = 3.2, w/b = 0.4) [37]	- Basalt: 3 mm, 6 mm, 12 mm, 18 mm in length. Vol. fractions: 0%, 0.1%.	Heat curing for 16 h.	The addition of 0.1% volume fraction of 6 mm basalt fibers in fly ash-based concrete led to the highest achieved splitting tensile strength of 4.84 MPa.
70% fly ash + 30% slag (NaOH + Na_2_SiO_3_, SiO_2_/Na_2_O = 1.5, w/b = 0.4) [26]	- Polypropylene fiber: 12 mm × 18–30 µm. Content: 0.1% to 0.5% - Basalt: 12 mm × 7–30 µm. Content: 0.1% to 0.5%. - Steel: 13 mm × 0.2 mm. Content: 0.1% to 0.5%.	Curing for 24 h and demold until 28 days.	The optimum values of fibers to enhance flexural strength were 0.2% polypropylene, 0.4% basalt, and 0.5% steel, which increased the flexural strength by 7.7%, 12.3%, and 21.5%, respectively.
60% fly ash + 40% slag (NaOH + Na_2_SiO_3_, NaOH:Na_2_SiO_3_ = 1:2.5) [41]	- Polypropylene: 12 mm × 40 µm: 0% to 5%.	Ambient curing.	The addition of 5% polypropylene fiber content into alkali-activated concrete containing 60% fly ash and 40% slag led to the highest increase in flexural strength (7.03 MPa).
60% fly ash + 40% slag (NaOH + Na_2_SiO_3_, SiO_2_/Na_2_O = 1.2, w/b = 0.28) [39]	- PVA: 8 mm × 40 µm: 0% to 2%. - PVA: 12 mm × 100 µm: 0.5% to 2%.	Standard curing. Values reported for 28 days.	The addition of 2% short PVA fiber content (8 mm in length) increased the flexural strength from 4.81 MPa to 13.45 MPa. Meanwhile, the addition of 2% long PVA fiber content increased the flexural strength to 18.19 MPa.
80% metakaolin and 20% class F fly ash (14 M NaOH + Na_2_SiO_3_, Na_2_SiO_3_/NaOH = 2) [31]	- Industrial steel: 10 mm in length. Tensile strength: 1225 MPa. - Waste tire steel fibers (WTSF): 10–60 mm × 0.3 mm.	Ambient curing.	Alkali-activated concrete incorporating both rubber particles and steel fibers exhibited higher flexural strength compared to the concrete reinforced solely with steel fibers, exhibiting enhanced post-peak energy absorption, owing to synergistic effects at the interfaces that allow for greater deformations.
100% slag (12 M NaOH + Na_2_SiO_3_, Na_2_SiO_3_/NaOH = 2.5) [90]	- Hooked-end steel: 50 mm × 0.8 mm. - Polypropylene: 48 mm × 0.85 mm: 0% to 1.5%.	Ambient curing for 28 days.	Addition of 2% polypropylene fibers increased the tensile strength from 3 MPa to 4.5 MPa. Meanwhile, the addition of 2% steel fibers increased the tensile strength to 4.3 MPa.
100% slag (8 M, 10 M NaOH + Na_2_SiO_3_) [114]	- AR-glass: 12 mm × 10–17 µm. - Basalt: 12 mm × 13–20 µm. - Polypropylene: 10–60 mm × 0.3 mm.	Air curing for 90 days after curing at a temperature of 60 °C.	Addition of 3 kg/m^3^ polypropylene, basalt, and glass fibers independently led to improvements in the fracture energy of slag-based concrete.
Slag (w/b = 0.45) fly ash (w/b = 0.27) fly ash + silica fume (w/b = 0.35) NaOH + Na_2_SiO_3_ [30]	- Hooked-end short steel: 12.5 mm × 0.4 mm: 0.3%, 0.6%. - Straight long steel: 50 mm × 1 mm: 0.3%, 0.6%. - Macro-polypropylene fibers: 54 mm × 0.8 mm: 0.3%, 0.6%.	Ambient cured at 20 °C.	Addition of 0.6% blended long and short steel fibers and macro polypropylene fibers in alkali-activated concretes did not improve splitting tensile strength, possibly due to the relatively low fiber content. However, slight improvements in flexural strength were observed, with steel fibers in alkali-activated concretes and macro polypropylene fibers.
100% slag (Ca(OH)_2_ + Na_2_SO_4_, w/b = 0.26, 0.3, 0.34, and 0.38) [93]	- Polyethylene: 18 mm × 12 µm. Vol. fractions: 0%, 1.75%.	Ambient curing for 28 days.	Addition of 1.75% of polyethylene fiber in slag-based concrete exhibited a tensile strength of 13.06 MPa and a tensile strain capacity of 7.50%. The composite with the lowest water-to-binder ratio of 0.26 exhibits higher toughness.
Slag + 10% cement + 10% sodium silicate (Na_2_SiO_3_, w/b = 0.4) [47]	- Steel: 10 mm × 0.03 mm: 0%, 0.5%, 1%. - Polypropylene: 10 mm × 0.012 mm: 0%, 0.5%, 1%. - PVA: 8 mm × 0.04 mm: 0%, 0.5%, 1%.	Sealed and water bath-cured specimens.	Water-cured concretes reinforced with 1% PVA or steel fibers experienced higher flexural strength than those sealed. This water curing enhanced the polymerization process and reduced porosity.
100% slag (Na_2_SiO_3_ + Ca(OH)_2_ w/b = 0.34, 0.38, and 0.44) [98]	- PVA: 12 mm × 39 µm. Volume fractions: 0%, 2%.	Ambient curing for 28 days.	Addition of 2% PVA fiber in slag-based concrete with the lowest water-to-binder ratio of 0.34 achieved the highest tensile strain capacity of 4.48%, first-cracking strength of 3.87 MPa, and tensile strength of 4.69 MPa at 28 days. The tensile strain-hardening behavior increased up to 4.5% ductility.
100% fly ash (NaOH + Na_2_SiO_3_, w/b = 0.4) [97]	- PVA: 18 mm × 12 µm. Vol. fractions: 0%, 2%.	Heat curing for 28 days.	Addition of 2% of PVA fibers in fly ash-based concrete cured at a temperature of 60 °C for 8 h resulted in the highest increase in tensile strength, reaching a value of 3.4 MPa.
100% slag (Na_2_SiO_3_ + Ca(OH)_2_) [100]	- PVA: 18 mm × 12 µm. Vol. fractions: 0%, 0.02%.	Air cured. Values are taken at 28 days.	Addition of 0.02% of PVA fiber in slag-based concrete increased the tensile strength to 4.7 MPa and tensile ductility to 4.3%.
100% fly ash (NaOH + KOH) [115]	- Carbon fiber: 0.24 mm in diameter. Vol. fraction: 39.47 ± 0.5%. - E-glass: 0.38 mm in diameter. Vol. fraction: 41.17 ± 0.3%. - Basalt: 0.16 mm in diameter.	Air cured at 70 °C for 24 h.	Carbon fibers outperformed E-fiber and basalt fiber in fly ash-based concrete in terms of increase in flexural strength and thermal conductivity at elevated temperatures. Carbon fibers exhibited strong adhesion to the geopolymer matrix, reducing pull-out. E-fiber showed volatilization and pull-out tendencies, and basalt fiber induced chemical reactions causing agglomeration.
100% fly ash (12 M NaOH + Na_2_SiO_3_) [33]	- Polypropylene: 12 mm × 0.0075 mm. Vol. fractions: 0%, 0.4%, 0.8%, 1.2%. - PVA: 8 mm × 0.04 mm: 0%, 0.4%, 0.8%, 1.2%.	Heat cured with 80 °C for 24 h. Values were taken at 7 and 28 days.	Addition of 1.2% polypropylene, steel, and polyvinyl alcohol fibers increased the flexural strength by 14.6%, 31.45%, and 39.84%, respectively.
100% fly ash, (8 M NaOH + Na_2_SiO_3_ Na_2_SiO_3_/NaOH= 2.5) [68]	- High-strength steel: 13 mm × 200 micron. - Polyvinyl alcohol (PVA): 8 mm × 40 micron. - Polypropylene: 12 mm × 50 micron. - Polyester fiber (PET): 6 mm × 30–40 micron. - Carbon: 10 mm × 15 micron.	Ambient curing for 28 days.	Increasing fiber volume fractions up to 1%, which was found to be the optimum level in the study, led to enhanced flexural strength and toughness in high-strength steel (HSS) and PVA-reinforced mortars. However, this enhancement was not significant in the case of CR, PET, and PP-reinforced geopolymer mortars.
80% OPC + 20% fly ash [116]	- Glass: 6–18 mm × 14 µm: 0%, 0.5% recycled coarse aggregates.	Ambient curing for 24 h.	The combined influence of glass fibers and fly ash in concrete has a more pronounced impact on its mechanical strength than their individual effects combined.
100% fly ash (Na_2_SiO_3_ + NaOH) [58]	Cellulose fiber.	Heat curing for 24 h.	Incorporating cellulosic fibers in geopolymers results in improved toughness, ductility, flexural capacity, and crack resistance compared to cement-based composites without fibers.
Fly ash + 20% slag (8 M, 12 M NaOH + Na_2_SiO_3_, NaOH/Na_2_SiO_3_ = 1, w/b = 0.4, 0.45) [17]	- Hooked-end steel: 35 mm × 0.55 mm. Vol. fractions: 0%, 0.5%, 1%, 1.5%.	Ambient curing for 28 days.	The flexural strength of the mortar increased with higher NaOH molarity and steel fiber content. The mixture containing 1.5% polypropylene fibers in 12 M NaOH-activated fly ash/slag concrete with a water-to-binder ratio of 0.4 achieved the highest flexural strength, reaching 9.8 MPa.
Slag + silica fume (8 M NaOH + Na_2_SiO_3_) [117]	- Cotton: 8 mm × 0.04 mm: 0%, 0.3%, 0.5%, 0.7%, 1%.	Ambient curing. Values taken at 28 days.	Addition of 0.5% cotton fibers improved the flexural strength, modulus, and toughness. However, exceeding 0.5% results in reduced flexural strength due to workability issues.
Metakaolin + fly ash (K_2_SiO_3_, SiO_2_/K_2_O molar ratios of 1.0) [101]	- Cotton: 6 mm × 7 µm. Vol. fractions: 2%.	Room temperature curing.	Addition of 2% cotton fiber increased the flexural strength. This increase happens slightly after exposure to 100 °C. It decreases in the range of 100–800 °C due to thermal deformations.
100% fly ash (10 M NaOH + Na_2_SiO_3_) [60]	- Sisal fiber: 35–40 mm × 179 µm: 0% to 1.0%. - Coconut: 35–40 mm × 117 µm: 0% to 1.0%. - Glass: 12 mm × 20 µm.	Heat cured.	Addition of sisal increased flexural strengths ranging from 5.3 to 6.6 MPa, outperforming the control mix. In addition, mortars reinforced with sisal fibers achieved splitting strengths between 2.2 and 3.3 MPa, outperforming the control mix.
100% fly ash (Na_2_SiO_3_ + NaOH) [76]	- Polyethylene: 12 mm × 20 µm. Vol. fractions: 0%, 1.5%, 2%.	Room curing followed by oven curing.	The incorporation of 1.5% polyethylene fiber increased the tensile strain capacity to 13.7% tensile strain capacity and tensile strength to 6.8 MPa after 28 days of testing.
GGBS + fly ash (8 M, 10 M NaOH +Na_2_SiO_3_, Na_2_SiO_3_/NaOH = 2.5) [18]	- Basalt: 12 mm × 13 µm.: Vol. fractions: 0.1% to 0.35%. - Crimped steel: 25 mm × 0.5 mm. Vol. fractions: 0.4% to 0.6%. - Polypropylene: 12 mm × 0.038 mm. Vol. fractions: 0.05% to 0.25%.	Ambient curing. Values were recorded for 7 and 28 days.	At both 7 and 28 days of curing with 8 M and 10 M NaOH concentrations, the inclusion of 0.55% steel fibers, 0.3% basalt fibers, and 0.1% polypropylene fibers independently led to the highest tensile and flexural strengths, with steel fibers showing the most reduction.
Cement + microsilica [32]	- Carbon fiber: 10 mm in length. Vol. fractions: 0.2%, 0.4%, 0.6%, 0.8%, 1%.	Values were recorded for 28 days of curing.	Addition of 0.6% carbon fiber content increased the tensile strength to 6.86 MPa. However, increasing the carbon fiber beyond 0.6% decreased the tensile strength.
100% OPC and OPC + 25% GGBS [102]	- Jute: 10 mm in length. Vol. fractions: 0.2%, 0.4%, 0.6%, 0.8%, 1%.	Water curing for 28 days. Then conditioned indoors for 7 days.	Addition of 0.5% jute fiber and superplasticizer increased the flexural strength by 24.3% and splitting tensile strength by 21%. When 0.5% jute fiber was combined with 25% GGBS and superplasticizer, it resulted in a 33% enhancement in flexural strength.
Metakaolin + fly ash (NaOH + Na_2_SiO_3_, Na_2_SiO_3_/NaOH = 6.27, w/b = 0.65) [40]	- PVA: 12 mm × 40 μm. Vol. fractions: 0%, 0.2%, 0.4%, 0.6%, 0.8%, 1.0%, and 1.2%.	Curing at 25 °C and 200 °C.	The inclusion of 1.2% PVA fibers led to a 58% and 66.3% increase in flexural strength at temperatures of 25 °C and 200 °C, respectively. As temperature exceeded 200 °C, significant decline in mortar flexural strengths was observed.
Metakaolin concrete (NaOH or KOH) [118]	- PVA: 12 mm × 40 μm: 0% and 2%. - Polyethylene: 12 mm × 20 μm: 0% and 2%.	Curing in dry plastic bags for two weeks.	The flexural strengths of NaOH-activated metakaolin concrete and potassium-based metakaolin concrete were measured at 19.7 MPa and 13.7 Mpa, respectively, while composites containing polyethylene fibers exhibited even higher flexural strengths.
Cement + GGBFS + fly ash + microsilica [19]	- Hooked-end steel: 35 mm × 0.55 mm: 0.5% down to 0.1%. - Microsteel: 10 mm × 0.12 mm.	Moist cured for 7, 14, and 28 days.	The combination of hooked steel and microsteel fibers with volume fractions of 0.4% and 0.1% led to 79.65% to 87.12% increase in compressive strength depending on concrete grade. There are similar improvements in flexural strength.
100% fly ash (NaOH + Na_2_SiO_3_) [35]	- Polypropylene: 12 mm × 34 µm. Vol. fraction: 0%, 0.05%, 0.1%, 0.2%.	Curing at 80 °C for 24 h.	The addition of 0.05% polypropylene microfibers enhanced the flexural strength by 17%. The toughness of geopolymer binders decreased with elevated temperatures exceeding 200 °C, particularly in fiber-reinforced specimens.
Slag + fly ash + silica fume (Na_2_SiO_3_ + NaOH, w/b = 0.32) [20]	- Corrugated steel: 13 mm × 0.12 mm. - Hooked-end steel: 13 mm × 0.12 mm: 0% to 3%. - Straight steel: 13 mm × 0.12 mm.	Standard curing at 20 °C.	Inclusion of steel fiber improves splitting tensile strength. Increasing steel fiber content to 3% leads to a substantial increase of 42.1% in splitting tensile strength.
Slag + fly ash + silica fume (Na_2_SiO_3_ + NaOH, w/b = 0.32) [20]	- Corrugated steel: 13 mm × 0.12 mm. - Hooked-end steel: 13 mm × 0.12 mm. - Straight steel: 13 mm × 0.12 mm.	Standard curing at 20 °C.	Increasing steel fiber content from 1% to 3% increased deflection corresponding to first crack, increased flexural strength, and decreased peak deflection.
Fly ash + slag + basalt powder [54] (Na_2_SiO_3_ + 12 M NaOH)	- Basalt: 12 mm × 0.02 mm. Vol. fractions: 0.4%, 0.8%, and 1.2%.	Cured at 60 °C for 24 h.	Addition of 1.2% basalt fibers led to a 34.15% increase in flexural strength. Even at 800 °C, the inclusion of basalt fibers led to improvements in flexural strength ranging from 11.61% to 44.69% when basalt volume fractions ranged from 0.4% to 1.2% compared to mortar without basalt.
Geopolymeric cement [10]	- Basalt: 45 mm and 9 µm. Vol. fractions: 0%, 0.5%, 1%.	Ambient curing for 1 day then water curing for 27 days.	Addition of 1.0% basalt fibers increased splitting tensile and flexural strengths in geopolymeric cement. Geopolymeric cement demonstrated superior load capacity and fracture toughness.
80% fly ash + 20% slag 50% fly ash 50% slag (Na_2_SiO_3_ + NaOH, Na_2_SiO_3_/NaOH = 2.5) [62]	- Fibers: coir, ramie, hemp, jute, and bamboo: 1% and 2% of the volume of fine aggregates: natural sand; lead smelter slag; and waste glass sand.	Ambient curing. Values taken at 7, 14, and 28 days.	Addition of 1% natural fibers enhances the tensile strengths of geopolymers. However, when coir, sisal, and jute fibers are added at the same percentage, there is a slight decrease in strength compared to unreinforced geopolymers. Geopolymers reinforced with 1% ramie fiber displayed the highest tensile strength.
100% slag (Na_2_SiO_3_ + NaOH, SiO_2_/Na_2_O molar ratio: 2.1) [119]	- AR-glass: 12 mm in length: 0% to 0.5%. - E-glass: 12 mm long. - Basalt: 12 mm long. - Carbon: 12 mm long.	Ambient curing at 20 °C.	Short fibers, particularly carbon fibers (content up to 0.5%), led to a substantial increase in flexural strength. This effect was observed both in the short term and over a longer duration of 28 days, resulting in 30% improvement in tensile strength.

**Table 5:** Summarizing studies on the durability properties of fiber-reinforced alkali-activated concretes and OPC sustainable concrete.

Binder Type	Fiber Characteristics and Content	Curing Age	Result
GGBS + 30% microsilica (Na_2_SiO_3_ + NaOH, Na_2_SiO_3_/NaOH = 3.5, w/b = 0.33) [12]	- Steel: 15 mm × 0.12 mm, 1% to 2.25%. - Polypropylene: 8 mm × 0.033 mm, 0.25%.	Ambient curing for 24 h	Mixes containing 2% steel fibers and 0.25% polypropylene fibers demonstrated the lowest chloride passing flow, resulting in a 47% reduction compared to the mix without fibers.
15% fly ash 85% OPC 20% fly ash 80% OPC [34]	- Polypropylene: Vol. fractions: 0.05%, 0.1%, 0.2%.	Ambient cured for 24 h then in water at 23 ± 2 °C	Porosity, water absorption, and sorptivity coefficient values increase with the increase in fly ash and fiber contents in all mixes, with fly ash having a more significant impact on the sorptivity coefficient than polypropylene fibers.
Fly ash + 25% RHA + 10% GGBS (Na_2_SiO_3_ + NaOH, Na_2_SiO_3_/NaOH = 2.5) [70]	- Basalt: 6 mm and 13–20 µm. Vol. fractions: 1%, to 4%.	Cured for 28 days.	Basalt fibers decreased water absorption rates and sorptivity of geopolymer concrete. This is attributed to the three-dimensional behavior of the basalt fibers, which helped block voids in 3D.
OPC concrete (w/b = 0.5) slag-based (Na_2_SiO_3,_ SiO_2_/Na_2_O = 1.18, w/b = 0.56) [55]	- AR-glass: 12 mm × 14–20 µm. Vol. fractions: 0.11%, 0.22%, 1.1%.	Air cured for 28 days.	The addition of 0.22% glass fibers to alkali-activated slag mortars resulted in a 20% reduction in drying shrinkage. This reduction was comparable to the shrinkage rate observed in Ordinary Portland cement mortar.
Slag + 10% cement + 10% hydrous sodium silicate (Na_2_O/SiO_2_ = 1, w/b = 0.4) [47]	- Steel: length/diameter (L/D) = 10/0.03: 0%, 0.5%, 1%. - Polypropylene: L/D = 10/0.012. - PVA: L/D = 8/0.04.	Sealed and water bath-cured specimens.	Sealed alkali-activated concrete without fibers had a water absorption rate of around 6.85%. The addition of 1% PVA fibers to the sealed concrete increased water absorption by 5%. The mixture reinforced with 0.5% PVA fiber and cured in a water bath exhibited the highest reduction in water absorption, exceeding 6%.
Slag-based concrete (Na_2_SiO_3_ + NaOH, Na_2_SiO_3_/NaOH = 2.5, 3) [14]	- Short length steel: 6 mm × 0.75 mm: 0%, 1.25%, 2.5%. - Long length steel: 12 mm × 0.75 mm: 0%, 1.25%, 2.5%.	Ambient curing and heat curing for 28 days.	Slag-based concrete containing short steel fibers showed lower permeability and void content, which restricted the ingress of water. Steel fibers enhanced resistance to sulfate exposure. Short steel fibers (6 mm) showed better results in enhancing the compressive strength compared to long steel fibers (12 mm).
Slag + 20% silica fume (Na_2_SiO_3_ + NaOH, SiO_2_/Na_2_O = 1.2, w/b = 0.17) [15]	- Brass-coated steel: 6 mm and 13 mm × 0.16 mm. Vol. fractions: 0.5%, 1%, 1.5%, 2%.	Heat-treatment cycles with steam curing at 100 °C for 12 h.	The inclusion of steel fibers decreased drying shrinkage by 24% compared to benchmark alkali-activated concrete. Both 6 mm and 13 mm steel fibers exhibited similar effectiveness in reducing drying shrinkage at the same fiber dosages.
OPC + 15% fly ash OPC + 20% fly ash [34]	- Polypropylene: Vol. fractions: 0.05%, 0.1%, 0.2%.	Ambient cured for 28 days.	Polypropylene fiber and fly ash contribute to a reduction in drying shrinkage, with the combination of the two leading to the lowest drying shrinkage. Freeze–thaw resistance of polypropylene fiber concrete is slightly higher compared to concrete without fibers, but fly ash enhances the freeze–thaw resistance.
100% fly ash (Na_2_SiO_3_ + 12 M NaOH) [33]	- Steel: 6 mm × 0.17 mm: 0.4%, 0.8%, 1.2%. - Polypropylene: 12 mm × 0.0075 mm: 0.4%, 0.8%, 1.2%. - PVA: 8 mm × 0.04 mm: 0.4%, 0.8%, 1.2%.	Heat cured at 80 °C for 24 h.	All types of fibers exhibited significant improvements in drying shrinkage compared to the control sample. A 1.2% PVA fiber content demonstrated the least drying shrinkage, followed by 1.2% polypropylene, and then 1.2% steel fibers.
Slag + silica fume (8 M NaOH + Na_2_SiO_3_) [117]	- Cotton: 8 mm and diameter of 0.04 mm: 0%, 0.3%, 0.5%, 0.7%, 1%.	Ambient Curing. Values taken at 28 days.	Cotton fibers decreased the density of the alkali-activated mortar. The higher water-to-fly ash ratios needed when more cotton fibers are included result in increased porosity and reduced density.
80% OPC + 20% fly ash [116]	- Glass: 6–18 mm × 14 µm: 0% and 0.5% recycled coarse aggregates.	Ambient curing for 24 h.	Glass fibers (GF) cause a slight increase in water absorption due to improved microchannel connectivity. Addition of fly ash reduces water absorption. The inclusion of GF led to a 5 to 11% increase in chloride penetration. The addition of fly ash mitigated the adverse effect of GF.
Cement + fly ash + slag (w/b = 0.41) [83]	- Polypropylene: 12 mm × 35 µm: 0.45 kg/m^3^, 0.9 kg/m^3^, 1.35 kg/m^3^.	Ambient curing for 24 h.	Chloride resistance is enhanced by the addition of polypropylene fibers. Chloride content in the polypropylene fiber concrete was reduced, with a maximum reduction of 34.6% observed when fiber weight is 1.35 kg/m^3^.
Fly ash + slag (8 M, 12 M NaOH, NaOH/Na_2_SiO_3_ = 1, w/b = 0.4 and 0.45) [17]	- Hooked-end steel: 35 mm × 0.55 mm. Vol. fractions: 0%, 0.5%, 1%, 1.5%.	Curing at room temperature for 28 days.	The inclusion of fibers in volumes ranging from 0.5% to 1.5% reduced chloride penetration depth and diffusivity and decreased the rate of strength loss when concrete is exposed to various chemical solutions for 120 days.
100% fly ash (12 M NaOH + Na_2_SiO_3_) [33]	- Steel: 6 mm × 0.17 mm: 0%, 0.4%, 0.8%, 1.2%. - Polypropylene: 12 mm × 0.0075 mm: 0%, 0.4%, 0.8%, 1.2%. - PVA: 8 mm × 0.04 mm: 0%, 0.4%, 0.8%, 1.2%.	Heat curing. Values were taken at 7 and 28 days.	Fibers reduced weight loss due to abrasion. The addition of fibers led to lower drying shrinkage values, with 1.2% PVA inclusion having the most positive impact, followed by 1.2% polypropylene fibers, and then 1.2% steel fibers, especially in the long term, where 1.2% PVA fiber-reinforced fly ash-based concrete reached a value of 400 macrostrains.
OPC + microsilica [32]	- Carbon: 10 mm in length. Vol. fractions: 0.2% to 1%.	Values recorded for 28 days of curing.	Carbon fibers enhanced concrete resistance to acid attacks and reduced mass loss after exposure to acidic environments. Carbon fiber prevented the formation and progression of cracks in the concrete when subjected to acid.
75% OPC + 25% GGBS [102]	- Jute: 10 mm in length. Vol. fractions: 0%, 0.25%, 0.5%.	Water curing followed by air curing then immersion in 10% NaCl solution.	Superplasticizer mitigated the adverse effects of fibers and decreased water absorption compared to the control mix after 28 days. Superplasticizer added to jute fiber-reinforced concrete led to a 17% reduction in chloride ion penetration depth for the 0.5% JFRC compared to the control mix.
94% cement + 6% natural pozzolan [135]	- Short polypropylene: 6 mm × 0.034 mm: 0%, 0.1%, 0.15%. - Long polypropylene: 12 mm × 0.034 mm: 0%, 0.1%, 0.15%.	Curing in tap water and sodium chloride water.	Addition of polypropylene fibers does not significantly alter the concrete’s ability to resist chloride ions penetration. The charge passed through high-performance concrete and high-performance fiber-reinforced concrete are comparable, particularly after 90 days of exposure.
Fly ash + slag + basalt powder (12 M NaOH + Na_2_SiO_3_) [54]	- Basalt fiber: 12 mm × 0.02 mm. Vol. fractions: 4%, 8%, and 12%.	Heat curing followed by air curing.	Increase in basalt fiber (BF) content led to higher water absorption. BF enhanced pore connectivity at higher volume fractions. The water absorption coefficient increased from 9.04% to 17.99% as BF content increased.
Slag + fly ash (Na_2_SiO_3_ + NaOH, SiO_2_/Na_2_O molar ratio: 2.1) [119]	- AR-glass fiber: 12 mm length: 0%, 0.5%. - E-glass: 12 mm long: 0% to 0.5%. - Basalt: 12 mm long. - Carbon: 12 mm long.	Ambient curing at a temperature of 20 °C for 24 h.	E-glass and basalt fibers (ranging 0% to 0.5% each) displayed similar patterns of deterioration, experiencing weight loss and a reduction in strength during alkali immersion. In contrast, AR-glass and carbon fibers (0% to 0.5%) demonstrated better durability under these conditions.
OPC + fly ash OPC + fly ash + silica fume [103]	- Basalt: 12 mm length. Vol. fractions: 0%, 0.15%, 0.3%, 0.45%, 0.6%.	Cured at 20 ± 1 °C.	Increase in basalt fiber content increased the electrical charge passing through the concrete as well as the chloride diffusion coefficient. Inclusion of mineral admixtures and the extension of the curing period mitigated the increased electrical charge/diffusion coefficient.
OPC [136]	- Basalt: 12 mm × 16 µm: 0%, 0.1%, 0.2%, 0.3%. - Polypropylene: 12 mm × 18–45 µm.	Cured at 20 ± 1 °C and ≥95% RH for 28 and 56 days.	Fibers decreased resistance to chloride penetration, with a more significant reduction with polypropylene fibers compared to basalt fiber. Extended curing alleviated the negative impact of fibers on concrete’s chloride resistance. Propylene fiber volume fraction: 0% to 0.3%
OPC + silica fume + fly ash + slag [104]	- Basalt: 18 mm × 15 µm: 0%, 0.05%, 0.1%, 0.15%. - Polypropylene: 19 mm × 30 µm: 0%, 0.05%, 0.1%, 0.15%.	Cured at 20 °C ± 2 °C and 98% relative humidity for 28 days.	There was a 77.8% reduction in the chloride diffusion coefficient of concrete containing a combination of 0.05% basalt fibers and 0.1% polypropylene fibers. When fiber volume fraction surpasses 0.15%, there is a gradual increase in the chloride diffusion coefficient.
